# Modelling acute myeloid leukemia (AML): What’s new? A transition from the classical to the modern

**DOI:** 10.1007/s13346-022-01189-4

**Published:** 2022-08-05

**Authors:** Annachiara Dozzo, Aoife Galvin, Jae-Won Shin, Santo Scalia, Caitriona M. O’Driscoll, Katie B. Ryan

**Affiliations:** 1 School of Pharmacy, University College Cork, Cork, Ireland; 2 Department of Pharmacology and Regenerative Medicine, University of Illinois at Chicago College of Medicine, 909 S. Wolcott Ave, Chicago, IL 5091 COMRB USA; 3 Università degli Studi di Ferrara, Via Luigi Borsari 46, 44121 Ferrara, Italy; 4 SSPC Centre for Pharmaceutical Research, School of Pharmacy, University College Cork, Cork, Ireland

**Keywords:** AML, cancer, Bone marrow, Preclinical models, Scaffold, Matrix, 3D model, Organ-on-chip, Biomaterials, Xenograft

## Abstract

Acute myeloid leukemia (AML) is a heterogeneous malignancy affecting myeloid cells in the bone marrow (BM) but can spread giving rise to impaired hematopoiesis. AML incidence increases with age and is associated with poor prognostic outcomes. There has been a disconnect between the success of novel drug compounds observed in preclinical studies of hematological malignancy and less than exceptional therapeutic responses in clinical trials. This review aims to provide a state-of-the-art overview on the different preclinical models of AML available to expand insights into disease pathology and as preclinical screening tools. Deciphering the complex physiological and pathological processes and developing predictive preclinical models are key to understanding disease progression and fundamental in the development and testing of new effective drug treatments. Standard scaffold-free suspension models fail to recapitulate the complex environment where AML occurs. To this end, we review advances in scaffold/matrix-based 3D models and outline the most recent advances in on-chip technology. We also provide an overview of clinically relevant animal models and review the expanding use of patient-derived samples, which offer the prospect to create more “patient specific” screening tools either in the guise of 3D matrix models, microphysiological “organ-on-chip” tools or xenograft models and discuss representative examples.

**Graphical abstract:**

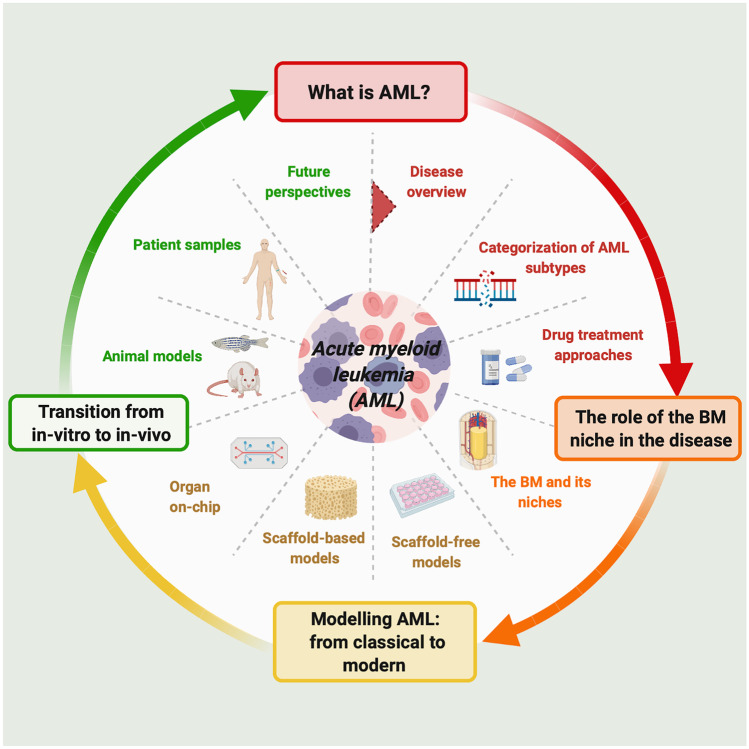

## Introduction

Leukemia is a type of blood cancer that can be classified considering the type of mutated precursor cell (e.g., lymphoid or myeloid) and how quickly the disease progresses (e.g., acute or chronic). Accordingly, leukemia can include acute lymphocytic leukemia (ALL), chronic lymphocytic leukemia (CLL), acute myeloid leukemia (AML) and chronic myeloid leukemia (CML) [1]. Myeloproliferative neoplasm and systemic mastocytosis are also classified as blood disorders but are rarer [2]. AML is a hematologic, heterogenous disorder caused by multiple cytogenetic and genetic abnormalities that occur in myeloid precursors [3–5]. Poorly differentiated myeloid cells can accumulate at their site of production in the bone marrow (BM) and spread in nearby blood vessels manifesting in impaired hematopoiesis and diverse symptoms including bleeding, bruising, infections, fatigue and bone pain [6, 7].

Age is an important factor and dictates prognostic outlook and treatment approaches. AML is more common in elderly populations, the median age at diagnosis is 68 years and it is less common in patients < 45 years [8, 9]. Population statistics confirm the aggressiveness of the disease. Patient 5-year relative survival rate is poor, around 28.3% [10, 11]. While the overall survival increases to 40–50% for patients < 50 years with de novo AML, it drops to 5–10% in elderly populations [12]. The American Cancer Society estimated that over 60,000 patients in America would be newly diagnosed with different types of leukemia in 2020 and of these, 19,940 would be diagnosed with de novo AML, predominantly in adults. While 11,180 patients were estimated to die from AML in 2020 [7]. Current treatment approaches include chemotherapy, radiotherapy, targeted biological therapies and stem cell transplantation. Treatment success is variable and can be further complicated by underlying health conditions especially in older patients and relapse rates are high [9].

AML arises in the bone marrow (BM), which is a complex environment consisting of regions called niches. Multipotent hematopoietic stem and progenitor cells (HSPC) which have the potential to differentiate and renew blood and immune cells reside in the stem cell niche [13]. These niches are defined by anatomy and function and described as local tissue microenvironments that maintain and regulate cells [14, 15]. In the BM niche, different environments include the endosteal, central and perivascular niches [16], and are described by different physical properties and constituents (e.g., proteins and cell types) [17, 18]. Hematopoiesis is tightly regulated and BM environmental cues including biochemical (cells, growth factors and cytokines) and physicochemical (stiffness, oxygen concentration, extracellular matrix) properties can regulate hematopoietic stem cells quiescence, activation and differentiation [13]. The function of the niches is subject to research and debate. Classically it is thought that the HSPC quiescence is maintained by the endosteal niche environment while the vascular niche regulates proliferation of short-term HSPCs; however other research has shown that dormant HSPCs reside in the perivascular niche in close proximity to blood vessels [19]. Therefore, the treatment of the uncontrolled proliferation and accumulation of abnormal, partially differentiated AML blasts and consequent impaired hematopoiesis, which are hallmarks of leukemogenesis [20], is further complicated by the BM environment. Emerging evidence points towards the involvement of niche constituents in driving neoplasia or undergoing remodeling to support AML cell survival and protecting residual AML cells after chemotherapy treatment, contributing to AML relapse [21, 22].

A better understanding of the multitude of factors impacting AML initiation and progression including cytogenetic and molecular variables have enhanced prognostic and treatment capabilities. This knowledge has also had a pivotal influence on the development and approval of multiple drug treatments towards the end of the last decade [23]. However, there are several factors still impacting the clinical development of potentially transformative medicines. Key amongst these is the availability of predictive preclinical models to evaluate the safety and efficacy of new therapies [24]. The development of physiologically relevant in vitro and in vivo models of AML and its environment (BM niche) will allow a better understanding of interactions between cancer cells and BM niche cells. Deciphering the complex physiological and pathological processes are key to understanding disease progression and fundamental in the development and testing of new effective drug treatments to target the niche and cellular components.

In the field of AML, preliminary and predictive drug screening tests have been carried out using cell suspension models; these together with 2D cell culture models have been very valuable in cancer research and drug discovery, representing the mainstay of in vitro models used [25]. These models fail to replicate the in vivo microenvironment and consequently cannot accurately predict drug performance in vivo. Studies have shown that the lack of biomimicry in 3D can impact on phenotypic behavior including response to drug treatments [26, 27]. Together with the knowledge that microenvironment can significantly impact the development and progression of many cancers [25], this has spurred interest in 3D culture models to recapitulate the biological environment more closely [28, 29]. Efforts directed towards reproducing the natural, intrinsic tissue cues using bioinspired materials and innovative fabrication techniques (e.g., 3D printing stereolithography) have been motivated by research in tissue engineering [30]. A wide variety of 3D matrix or scaffold-based approaches and models inspired by organ-on-chip technology have been investigated to more closely mimic the 3D environment where cells grow in terms of composition or physicochemical cues, e.g., hypoxia, chemical signaling gradients and fluid shear forces [31]. This is envisioned to enable more accurate screening of novel therapies and progression of promising candidates to further preclinical evaluation using animal models. Indeed, this has been proposed to bridge the gap between more primitive cell culture experiments and complex small animal models in addition to providing insights into disease progression that may be difficult to discern in animal models [25].

This review focuses on gold standard models used in AML research, their advantages and limitations. We review in vitro suspension cell culture models and the cell lines employed. Murine in vivo models represent a mainstay approach in AML research, however other useful models have also emerged including zebrafish. We explore the increasing use of more advanced cell culture models, which encompass 3D biomimetic scaffolds and organ-on-chip technologies in an effort to more closely replicate and physiologically model the native microenvironment and address the limitations of traditional cell culture models. As part of this, we focus on the materials and cells used to simulate the BM microenvironment. We also examine the increasing focus on patient derived samples which represent valuable preclinical screening tools in the context of personalized applications.

## Acute myeloid leukemia

### AML: Origins, categorization and diagnosis

AML is heterogeneous in terms of disease etiology. Its diversity is exemplified by differences in pathophysiology, clinical, cytogenetic and molecular profiles [23]. The World Health Organization (WHO) classification system (2016) has established several categories including AML with recurrent genetic abnormalities, AML with myelodysplasia related changes, AML owing to previous chemotherapy or radiation, AML types that are not categorized by other groups, myeloid sarcoma, myeloid proliferations related to Down syndrome and mixed phenotype leukemias, although this latter category is not strictly considered AML [32, 33]. It can arise due to previous treatment, although it occurs predominantly as a de novo malignancy and most cases are not attributed to inherited genetic defects. A review published in the New England Journal of Medicine highlighted the genetic diversity inherent in AML. Among 1540 patients enrolled, 5234 driver mutations across 76 genes or genomic regions were identified, with 86% of patients presenting with two or more drivers [34]. In general, 55% of all adult patients with AML show prognostic cytogenetic abnormalities that can be classified by a wide range of chromosomal alterations [35]. Examples of recurrent cytogenetic entities implicated include t(8;21)(q22;q22.1); *RUNX1/RUNX1T1*, inv(16)(p13.1q22) or t(16;16)/ (p13.1q22) *CBFB-MYH11*, and acute promyelocytic leukemia (APL) t(15;17)(q22,q21) [23, 33]. Many AML patients show normal karyocytes [36, 37], but they acquire activating de novo mutations that have prognostic relevance and impact on many biological functions, including length mutations or internal tandem duplications of the *FLT3* gene (*FLT3*-ITD) [35, 38, 39] and mutations of transcription factor genes, such as *CEBPA,* which plays an important role in differentiation [40, 41]. Other relevant mutations include the nucleophosmin-1 (*NPM1*) gene (40–50% normal karyotype AML) [42], and isocitrate dehydrogenase (IDH) 1 or 2 (IDH 1/2) enzymes, which are prevalent in about 20% of AML [23].

Adopting the best treatment approach starts with an accurate diagnosis and understanding of disease pathophysiology in the case of each patient. Currently, there are many different methods used in the diagnosis of AML. Earlier approaches to define AML subtypes were based on the French, American and British (FAB) nomenclature system and involved metrics based on cell counting and microscopic analysis of cell morphology of the blast populations contained in blood and BM aspirate samples. Stratification into sub-types M0 to M7 is based on cell type (red, white cell or platelet) and maturity [43], although does not take account of the relative impact of different cytogenetic and genetic abnormalities. A shift in classification based on morphology to systems taking account of causative genomic changes is reflected in the WHO classification system, which as outlined above includes a category on AML with recurrent genetic abnormalities [34]. This system has been readily adopted and important in helping to refine prognostic subgroups and specify appropriate treatment strategies [44]. The European LeukemiaNet (ELN) stratifies the risks associated with different genetic factors as favorable, intermediate, and adverse and is widely used in practice to guide prognosis and management of AML [33]. For example, mutations on *NPM1* are associated with a more favorable prognosis, although their association with *FLT3*-ITD diminishes the outlook [33, 35].

According to the WHO classification (2016), the threshold for AML diagnosis is the presence of 20% or more of blasts in bone marrow or peripheral blood. Although a diagnosis can be made in patients with < 20% blasts who have recurrent cytogenetic abnormalities t(15;17), t(8;21) t(16;16) or inv(16) [44]. Diagnosis and accurate disease categorization relies not only on analysis of cell morphology but requires multiple, reliable diagnostic techniques including immunophenotyping using flow cytometry and immunohistochemical staining and genetic analysis to identify both translocations and gene mutations [45]. Common antigens expressed by AML cells include CD25, CD32, CD33, CD44, CD47, CD96 and CD123, with CD33 being expressed in the majority (85–90%) of AML cases [46–48]. In one study, 99 cases were examined and CD33 was expressed in 90% of the samples assessed [49].

### Treatment approaches

Treatment strategy is considered based on both disease specific and patient-related factors. Disease subtype, patient’s overall health status and personal preferences with respect to quality of life are key considerations [50]. As outlined, multiple malignant cell clones exist in most patients, and genetic and epigenetic variability in each patient can impact prognosis and treatment response [51]. Acute promyelocytic leukemia (APL) is regarded as a medical emergency and treated differently. Age is an important factor in stratifying patients for treatment and understanding risk profile. Patients are categorized as (i) aged between 18 and 60 years of age, (ii) 60 years old and (iii) older patients, over 74 years of age with co-morbidities [3].

Generally, long established AML chemotherapeutic regimens consist of a two-phase approach. The first phase, the “induction phase” involves treatment with intravenous (IV) chemotherapy drugs [33]. Typically, the “7 + 3” chemotherapy regimen has been the standard of care. This therapeutic regimen provides the daily administration of cytarabine in the first week, in association with an anthracycline, e.g., daunorubicin, idarubicin or mitoxantrone for the first 3 days [33, 52]. In cases of patients carrying specific gene mutations, the therapy can be enforced with a third drug such as midostaurin (FLT3 positive) [53] or gemtuzumab ozogamicin (CD33 positive). It is estimated that this combination of drugs is successful in 55–65% of patients < 60 years, with remission achieved. However this figure is lower (25–50%) in older patients [54].

Clinically, remission corresponds to a blast count less than 5%, otherwise the patient needs further therapy [55]. Depending on their risk profile and age, patients can either follow a more supportive chemotherapy treatment to further consolidate the induction phase. Alternatively, they undergo stem cell transplantation [56–58]. In severe cases, more aggressive chemotherapy regimens may be employed. Newly diagnosed elderly patients (> 75 years of age) or those with co-morbidities may not be able to withstand harsh chemotherapy regimens and alternative protocols for these patient cohorts are required [9, 59]. Recommendations for diagnosis and management of AML have been developed by an international expert panel on behalf of the European LeukemiaNet (ELN) [33]. The recommendations provide an overview of selected care regimens for patients eligible for intensive chemotherapy and options for those not considered candidates for intensive chemotherapy.

Recent advances in AML treatments are based on knowledge of the cellular mechanisms of the disease, cell cycle and enzymes involved, clonal expansion, and microenvironmental cues involved in the stem cell niche [23]. Important developments can be linked to the identification of the type of aberrancy in cellular DNA or targeting of surface markers [60]. Novel approaches in therapy involve de novo discovery of drugs or an evolution of the previously established treatments but with ameliorated formulation design, selective targeting, and decreased side-effects [33]. The novel liposomal formulation (Vyxeos^®^) is clinically used in the 7 + 3 regimen and combines the delivery of two drugs in a fixed molar ratio of 1:5 (daunorubicin to cytarabine) to give a synergistic effect and, to sustain the drugs in the BM for a prolonged period (for 24 h post administration) and assure preferential uptake by leukemic cells. The formulation has shown increased remission rates, survival and overall hematologic recovery in older patients (60–75 years) in a Phase III clinical trial which compared Vyxeos^®^ to the standard 7 + 3 induction regimen [61, 62]. However, the myelosuppression induced with the treatment was observed to last longer [63].

In the last few years, progress in translating novel therapies to the clinic has expanded the repertoire of treatments available. New and emerging agents include small molecule inhibitors, e.g., glasdegib, an inhibitor of the SMO (smoothened) surface protein [64], and IDH-1 and IDH-2 inhibitors, ivosidenib and enasidenib. Another class that belongs to the family of “mutationally targeted inhibitors” include FLT-3 inhibitors. Midostaurin has been clinically approved, while a number of others are undergoing trials [63]. Other therapeutic approaches include the use of pro-apoptotic agents such as venetoclax either in single or in combination with hypomethylating agents azacitidine or decitabine [65]. Other pro-apoptotic agents include BCL-2 inhibitors, while Mouse double minute 2 (MDM2) and P53 are other targets of pro-apoptotic agents [66]. Alternatives are offered by blocking the cell cycle checkpoints with Aurora kinase inhibitors, e.g., barasertib or blocking PLK-1 (polo-like kinase), and CDK (cyclin dependent kinase) [63]. Onvansertib (PCM-075) is a third generation, orally active, selective ATP-competitive PLK1 inhibitor which has displayed anti-tumoral activity both in vitro and in vivo against hematologic malignancies and other solid cancers [67–69]. A current Phase 1b/2 study (NCT03303339) is investigating onvansertib in combination with either low-dose cytarabine or decitabine (phase 2) in subjects with AML [70, 71].

Another option to target AML involves treatments that disrupt the microenvironment in which the malignant cells reside, e.g., uproleselan is a selective antagonist of the adhesion molecule, E-Selectin [72, 73]. Other approaches involve anti-angiogenic therapies and CXCR4 and CXCL12 antagonists. Some chemotherapy regimens may benefit from the addition of other agents. All-trans retinoic acid (ATRA) ligands on the endogenous retinoid receptors have been found to promote apoptosis by inhibiting the expression of Bcl-2 in AML cells [74].

Chemotherapy has remained the gold standard for a long time, but there is increasing interest in more targeted therapeutics [75], and together with advances in immunotherapy these are being increasingly exploited to target AML, in a bid to harness the immune system to enhance treatment efficacy and preserve healthy cells [76, 77]. In particular, the prevalence of the CD33 antigen together with the fact that high levels of expression in childhood leukemia correlates to adverse disease outcome has focused attention on CD33 as an important immunotherapy target [78–80]. Current therapeutic approaches include the antibody–drug conjugate Gemtuzumab Ozogamicin (GO) (Mylotarg^®^, Pfizer USA), which contains calicheamicin, a cytotoxic agent [81, 82].

The field of AML treatment is broad and includes multiple approaches. In addition to antigen-based strategies that bind to leukemic cells, other more novel immunotherapy approaches include the BiTE, bispecific T-cell engager technology, AMG 330 (CD33 + CD3)[63], which binds to CD33 on the tumor cell and CD3 on the T-cell. This technology demonstrated promising anti-leukemic activity in Phase I studies to target relapsed and refractory AML by exerting cell lysis [83, 84].

An unconventional and innovative approach to AML treatment has focused on vaccines to stimulate an immune response by targeting AML-antigens including Wilms Tumour protein 1 (WT-1) [85, 86]. Shah et al. recently developed a biomaterial-based “cryogel” vaccine to produce a more robust and durable immune response [87]. The macroporous scaffold, consisting of crosslinked methacrylated PEG (MA-PEG) and methacrylated alginate (MA-alginate), was used to deliver granulocyte–macrophage colony stimulating factor (GM-CSF), the Toll-like receptor 9 agonist, cytosine-guanosine oligodeoxynucleotide (CpG-ODN) and one or multiple AML antigens, e.g., WT-1 to activate the immune response against leukemic cells, via activation of dendritic cells. In vivo testing in mice demonstrated a potent anti-AML response and prophylactic administration prevented AML cell engraftment. Testing in a therapeutic disease model in combination with the cytotoxic induction chemotherapy regimen (cytarabine (Ara-C) and doxorubicin) eradicated the disease. The research highlights the potential of therapeutic vaccines to achieve lasting AML-specific immunity and address the problem of residual disease that does not succumb to chemotherapy treatment [87].

Novel gene therapies represent enormous potential, especially where increased specificity for AML cells can be achieved, thereby increasing efficacy and reducing side-effects. However, the therapeutic use of nucleotides (e.g., siRNA) is challenging owing to the high molecular weight, surface charge and half-life (30 s), which act as barriers to delivery [48, 88, 89]. Potential strategies to overcome the aforementioned problems, which have already been employed in the treatment of other diseases, include the use of non-viral delivery vehicles e.g., lipid-based nanoparticles used in ONPATTRO^®^ (Patisiran) a product marketed by Alnylam to treat liver disease. While the covalent conjugation with the targeting ligand N-acetylgalactosamine (GalNAc), which has guaranteed the conjugate long-term activity weeks after dosing, has been employed in several siRNA products translated to the clinic including GIVLAARI^®^ [90].

### Anatomy of the bone and the bone marrow

**Fig. 1:**
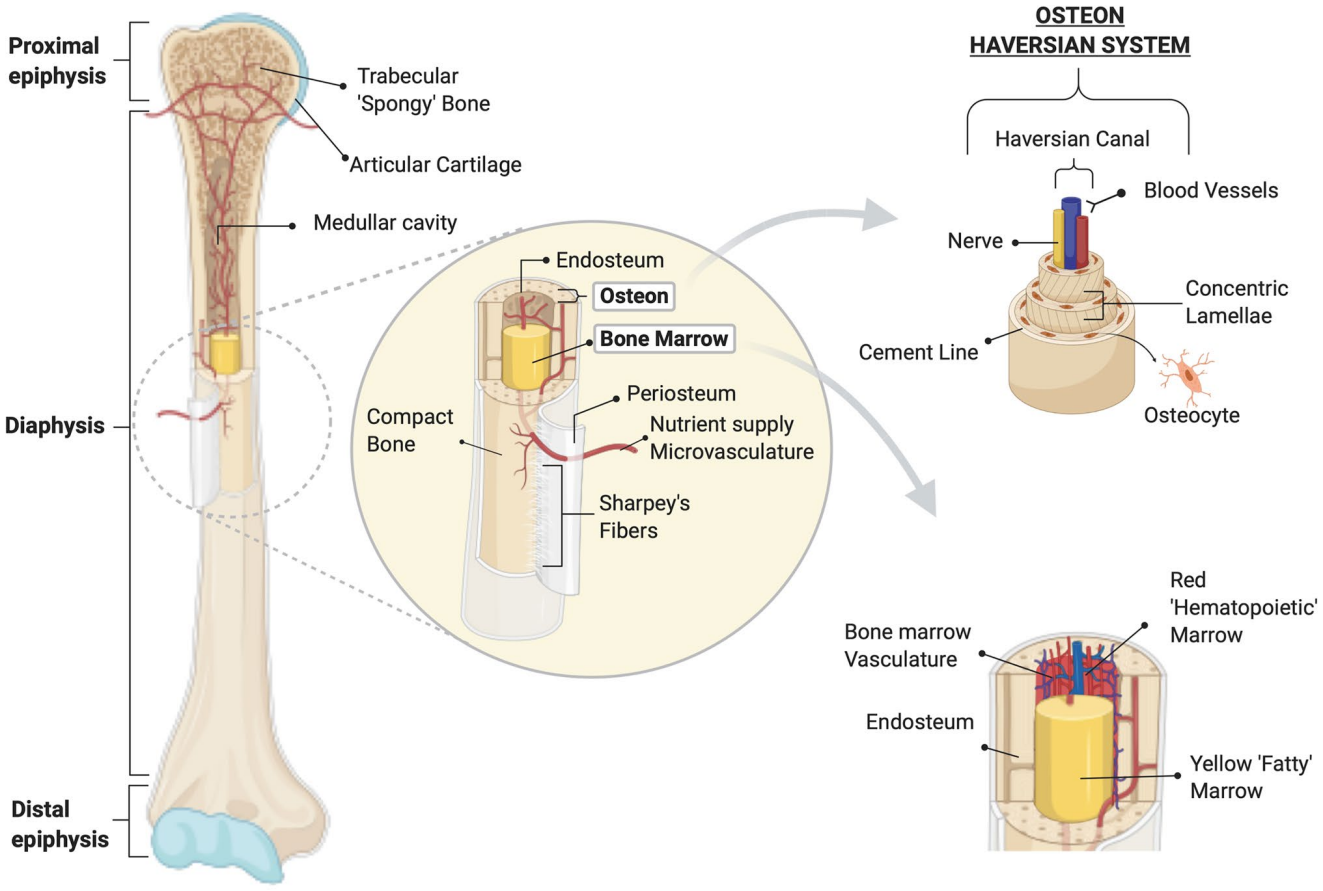
Schematic representation of the femur bone and relative inner hierarchical structure. **(Left)** Long bones such as the femur consist of two main parts: the parts at the top and bottom, are called the proximal and distal epiphysis, respectively, and a central tubular one called diaphysis. (**Center)** The cross section of the femur at the diaphysis shows the different layers the bone is composed of. The outer layer is the periosteum which covers much of the bone structure and is anchored to it through perforating fibers called Sharpey’s fibers. The inner layer is the endosteum at the boundary with the marrow cavity. **(Top Right)** Small round tubular lamellar units termed Haversian systems or osteons run longitudinally along the bone length in long bones. The typical rigidity and mechanical strength they confer on the bone depends on the different orientation of the concentric lamellae. The cavity in the osteons known otherwise as the Haversian canal, hosts nerve and blood vessels for nutrient supply. **(Bottom Right)** Bones hosts two types of bone marrow: the ‘red’ and the ‘yellow’ marrow which are differently specialized. The ‘red’ or ‘hematopoietic’ marrow is where hematopoiesis occurs and surrounds externally the ‘yellow’ marrow which fills the hollow part of the marrow cavity. The ‘yellow’ fatty marrow is rich in adipocytes but also contains MSCs. Created with BioRender.com

It is commonly known that the human body is composed of more than 200 bones [91, 92]. It is an exceptional tissue capable of self-repair and fulfills numerous functions including support, facilitation of movement, protection of vital organs, mineral homeostasis and hematopoiesis within the marrow space [93–96]. Anatomically, bones consist of two different tissue structures, cortical and cancellous bone, otherwise known as compact and trabecular bone. Both are similar in composition comprising of organic extracellular matrix (ECM) that predominantly consists of collagen and inorganic, carbonated hydroxyapatite-like mineral components. However, they differ in their architecture and the amount of matrix deposited. Cancellous bone is lower in density and consists of a supporting network of trabecular struts, which explains its porous structure. It is located centrally in long bones, mainly at the epiphysis and metaphysis, and is in close contact with the BM that fills its trabeculae [97, 98]. Cortical bone, is a dense layer, and its rigidity derives from the tightly packed cylindrical-shaped units called Osteons or Haversian systems that consist of concentric lamellae of bone matrix (Fig. 1) [97, 99]. The higher density in cortical bone accounts for its increased mechanical properties compared to cancellous/trabecular bone. Each Haversian system is separated from the surrounding tissue by a physical boundary called the cement line. The Haversian systems contain a central canal, which carries blood vessels and nerves [100, 101], and they are connected with each other through the Volkmann canals [100]. The periosteum, the envelope on the bone surface, consists of two layers, a superficial fibrous, thicker layer and the deeper cambium layer, which consists of undifferentiated MSCs, osteoblasts (OBs) and fibroblasts [102–104]. The endosteum consists of a thin layer of connective tissue that lines the cavity of long bones [91].

Bone cells represent at least the 10% of the entire volume of the bone and 4 key groups may be distinguished based on their morphology and function [92, 105]. Undifferentiated MSCs and progenitors reside in the BM but an exact location is debatable and difficult to trace as they may migrate to other sites [106]. MSC may be found in the vasculature or on the endosteal surface of the trabecular bone, the periosteum and bone canals [17, 107, 108]. MSCs are irregular shaped cells with an intrinsic potential to differentiate into osteoblasts (OBs) or adipocytes depending on environmental cues [109]. OBs are cuboidal shaped cells, and are characterized by abundant endoplasmic tissue, Golgi apparatus and mitochondria [91, 110]. They play an important role in synthesizing new bone matrix by mineralizing the dense collagen fiber network [111, 112]. They are also involved in regulating electrolyte homeostasis between the extracellular fluid and the bones, but under the influence of the parathyroid hormone they may activate osteoclasts [105]. OBs can follow different fates. They can differentiate into bone-lining cells that reside on the surface of the bone [113]. Bone lining cells exhibit morphological and phenotypic differences compared to OBs, for example they express vascular adhesion molecule 1 but do not express osteocalcin [114, 115]. They play a role in bone remodeling and the differentiation of osteoclast precursors into osteoclasts [116, 117]. Terminally differentiated OBs can become embedded in the mineralized bone environment and are termed osteocytes. They account for approximatively 90–95% of the total cells in bone and are thought to be important in bone remodeling [118, 119]. They exhibit different morphologies depending on the site in the bone where they reside [103], and can also be influenced by pathological states, e.g., osteoarthritis or osteoporosis [120, 121]. Alternatively, OBs can undergo apoptosis [122].

Osteoclasts are multinucleated cells originating from HSCs through the myeloid pathway [123, 124]. They are capable of resorbing both the mineral and proteinaceous components of bone by acidifying the local environment and secreting proteolytic enzymes including (matrix metalloproteinases) MMPs [125–127]. In their active state they reside in specific resorption cavities, called Howship's lacunae [91]. They exhibit a typical “wavy” cytoplasmic membrane otherwise called the “sealing zone”, which helps them to adhere to the matrix and operate its degradation [128]. The processes of bone formation and resorption are closely linked in bone remodeling. Osteoclastogenesis, the osteoclast forming process, is driven by binding of receptor activator of nuclear factor-kappa B ligand (RANKL) at the RANK receptor. RANKL is secreted by bone marrow stromal cells, OBs or osteocytes [129], and is attenuated when osteoprotegerin, a soluble decoy, competitively links with RANKL preventing binding to the RANK receptor [130]. The presence of macrophage colony stimulating factor (M-CSF) stimulates osteoclast proliferation and survival.

## Pathophysiology of acute myeloid leukemia

### HSC niche

**Fig. 2:**
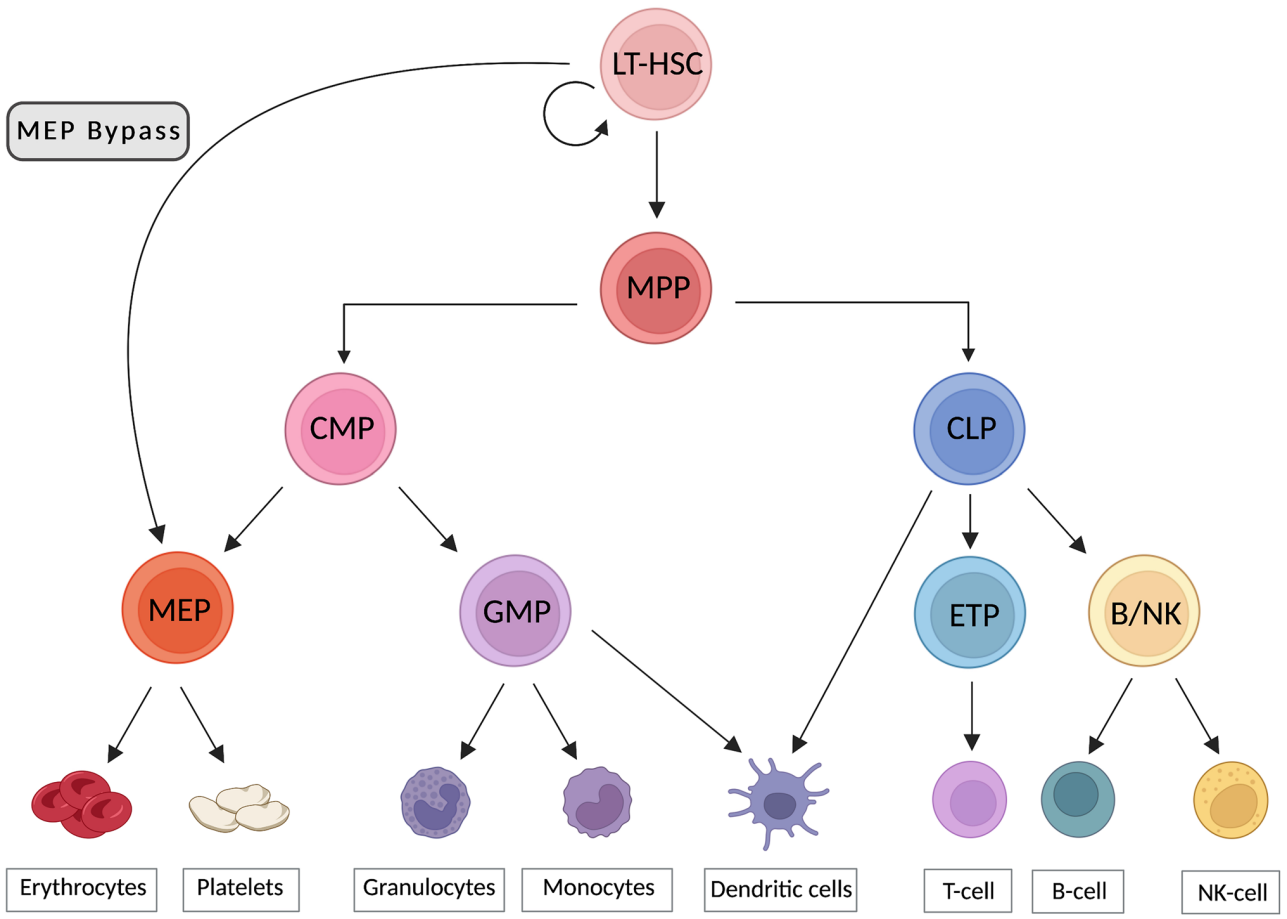
Revised model for human HSC hierarchy. In the classic model for the human HSC hierarchy long-term hematopoietic stem cell (LT-HSCs) sit at the top of the hierarchy and differentiate into multipotent progenitors (MPP). Downstream of MPP, separation into common myeloid progenitors (CMP) and common lymphoid progenitors (CLP) occurs. CMP can generate granulocyte-monocyte progenitors (GMP) and megakaryocyte-erythrocyte progenitors (MEP), while lymphoid progenitors form T, B, NK and dendritic cells. Further GMP differentiate into granulocytes and monocytes and MEPs generate megakaryocytes and erythrocytes. In a revised model, HSCs can differentiate directly into MEP by bypassing CMP (here represented as MEP bypass route). Redrawn and modified from Tajer et al. [135] under the terms of the CC BY 4.0 creative commons licence http://creativecommons.org/licenses/by/4.0/

The BM is a soft gelatinous tissue and occupies the central cavity of bones and is comprised of red and yellow marrow [131]. The adipocyte-rich yellow marrow is typically found in the long bones [132]. The red marrow is highly vascularized and localized on the endosteal surface of long bones and generally surrounds the yellow marrow, although it is more likely to be found in flat bones (e.g., skull, ribs), and contributes to hematopoiesis [131]. The red BM is the principal site for multipotent HSCs, which have a capacity for self-renewal and differentiation and give rise to common myeloid progenitors (CMP) and common lymphoid progenitors (CLP) that eventually reconstitute mature components of the blood and immune system including erythrocytes, myelocytes, platelets and lymphocytes [133], Fig. 2. Stepwise differentiation is controlled by key transcription factors and cytokines [134].

**Fig. 3:**
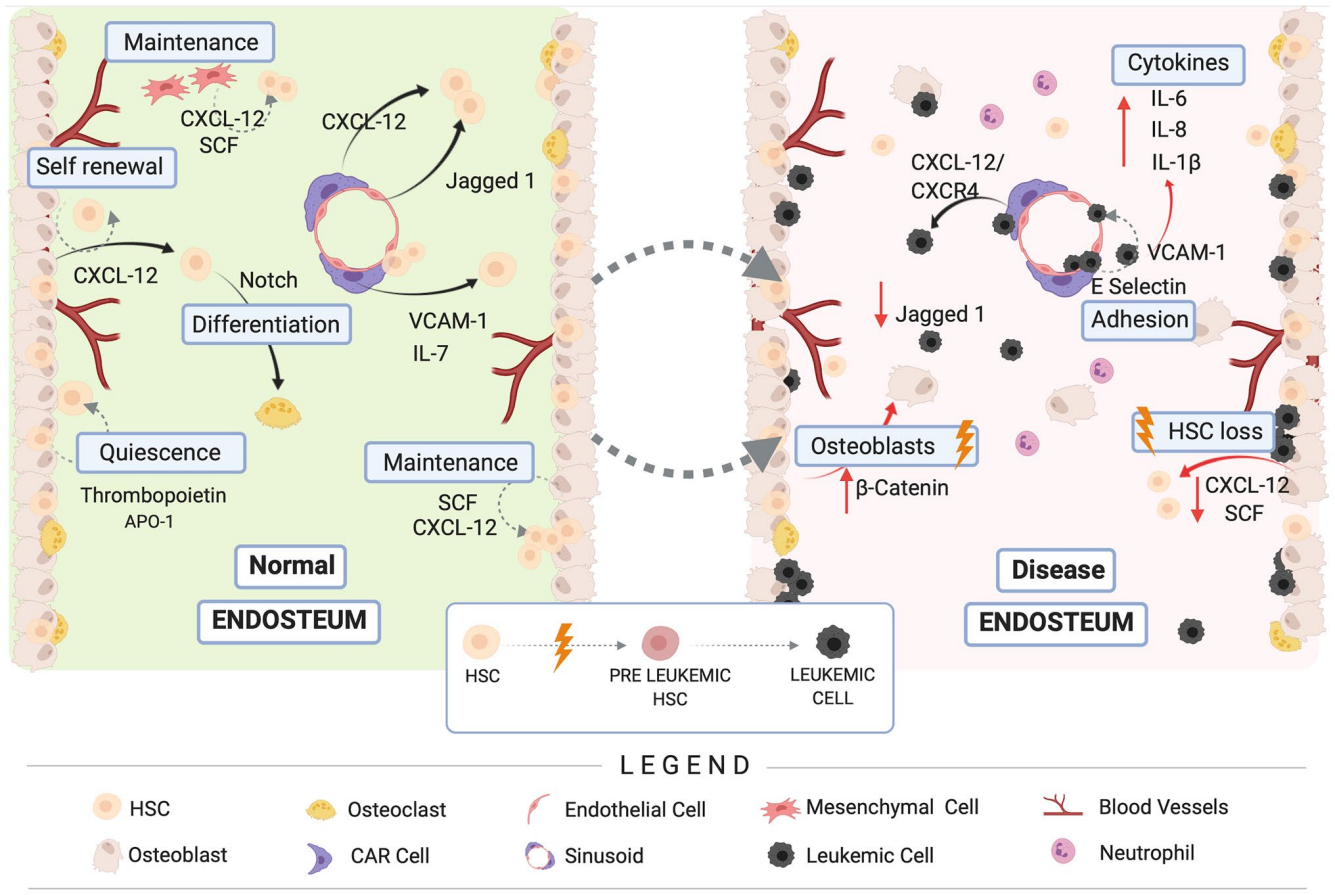
Schematic illustration of the cells involved in the maintenance of the red BM niche with simplified representation of the interplay occurring in healthy and leukemic states. HSCs reside mainly within the BM and frequently localize adjacent to blood vessels. In healthy marrow (left), some of the HSCs are in a quiescent state, some others differentiate or operate self-renewal for repopulation. Many different cells either hemopoietic and non-hemopoietic in origin coexist with the HSCs in the BM niche and actively take part in their maintenance, differentiation, and self-renewal processes. Osteoblasts initially were thought to regulate HSC maintenance via CXCL-12 and SCF but recently MSCs were found to be implicated. When leukemogenesis occurs (right), the resulting effects cause the disruption of cell molecular signaling pathways in the niche. The altered BM microenvironment offers the leukemic cells protection and contributes to the development of the disease. Leukemic cells adhere to the endothelium through soluble adhesion factor E-selectin and VCAM-1. CAR cells offer protection to leukemic cells via CXCL-12/CXCR4 interaction. An inflammatory state initiates in the BM and soluble cytokines (e.g., IL-6, IL-8, IL-1β) are released. Loss of the HSC pool happens through reduction of CXCL-12 and SCF. Created with BioRender.com

The concept of the HSC niche was first introduced by Schofield [136], and identifies a site, in the BM, where the HSC are retained in a quiescent state to protect their genomic integrity and functionality and operate self-renewal [137–139]. This concept is still debatable since there is no real boundary in the BM and HSCs can freely move, but can be localized through the cells the HSCs interact with (e.g., endothelial cells, MSCs or osteoblasts (OBs) and osteoclasts), which line the surface of the endosteum or the trabecular regions of bone [17, 133]. To ensure the homeostasis and the replenishment of the blood cells on a daily basis and after an injury, HSCs undergo differentiation. Niches are complex, dynamic environments influenced by a variety of factors including cell components, secreted factors, ECM and physical properties [140]. HSCs interact with various niche components, Fig. 3 to regulate their proliferation, differentiation and homing process to the BM [133]. However, a complete picture of the molecular interactions controlling HSC fate is still unclear [141].

A number of non-hematopoietic (e.g., MSCs, adipocytes and glial cells) and HSC derived (e.g., megakaryocytes, macrophages, T-cells) cells have been found to directly or indirectly regulate HSC in multiple ways which involve the secretion of factors such as CXC-chemokine ligand 12 (CXCL12), otherwise known as stromal derived factor-1 (SDF-1), and stem cell factor (SCF) among many others [22]. Osteoblasts, for example, were originally thought to be implicated in HSC maintenance through CXCL12 and SCF and quiescence via thrombopoietin and angiopoietin 1. However, more recent findings have postulated that OBs do not directly influence or regulate the HSCs based on the fact that deletion of those factors has very little effect on HSC activity [142]. It seems more likely that other cells like MSCs are involved in the maintenance of the HSC pool. This possibility has been supported by the finding that MSCs express a *Nes*-GFP transgene and HSCs are found in the vicinity of *Nes*-GFP^+^ cells which also produce CXCL12 and SCF. Endothelial cells (ECs) also contribute to the maintenance of HSCs and some of the first evidence to support this involved studies where the deletion of the cytokine receptor gp130 in endothelial cells led to the reduction of HSCs [143]. Many other factors like angiopoietin, IL-7, vascular cell adhesion molecule 1 (VCAM-1) and Jag1, which are also released by ECs have been discovered to influence HSCs and the depletion of those factors has been found to directly impair their maintenance [22].

Leukemogenesis, is linked to the dysregulation of molecular and cellular processes giving rise to leukemic stem cells (LSCs), which progressively impact the myeloid differentiation process [144]. This is characterized by uncontrolled proliferation and accumulation of an abnormal population of partially differentiated AML blasts [20]. Leukemic stem cells share some features with healthy HSCs; therefore it has been thought that they similarly reside in the BM niche, depending on it for proliferation and survival [145]. Indeed, LSC can remain in a quiescent state and together with treatment resistant cells can contribute to disease relapse. Among the different reasons that contribute to the development of de novo AML, genetic mutations, and reciprocal collaboration with other cells in the marrow niche can be of interest. Endothelial cells are known to actively contribute to leukemogenesis and the co-culture of ECs with AML cells has resulted in increased proliferation and exacerbated the AML cells malignant phenotype [146]. Many cytokines and other soluble factors including IL-6, IL-1β and IL-8 have been directly implicated in the mutual crosstalk established between leukemic and niche cells [147].

Leukemogenesis causes the disruption of cell molecular signaling pathways in the BM niche. The altered BM microenvironment offers the leukemic cells protection and contributes to the development of the disease. For example, AML cells exhibit the ability to adhere to stromal cells which results in a marked reduction in chemosensitivity [148]. It seems that the BM niche is altered by leukemic cells that also stimulate the expression of CXCR4 and very late antigen (VLA)-4, which promote cell adhesion [22]. The interaction between AML cells, VLA-4 and stromal fibronectin is a decisive factor for AML minimal residual disease [148]. The over expression of E-selectins, could also partially explain the chemoresistance and the high rates of relapse for this disease. One of the mechanisms used by AML cells involves positive interaction with ECs in the vascular niche to ensure protection [149]. High levels of E-selectins produced by ECs in a mouse model, were also related to the inflammatory state induced by AML cells in the BM. It has been later theorized that the overexpression of specific factors by ECs may be less involved in the homing of AML cells but contributes to their survival by creating a protective niche for LSCs [150].

## Models of acute myeloid leukemia

Current preclinical models of AML comprise a broad list including in vitro (e.g., 3D scaffold-free, 3D scaffold/matrix based, on-chip) models, in vivo non-mammalian (e.g., Drosophila or Zebrafish) and mammalian (e.g., murine) models [151] and ex vivo models, in the case of samples donated by patients.

### In vitro scaffold-free models of acute myeloid leukemia

Standard scaffold-free models include suspension cultures where AML cells or other types of suspension cells can grow and interact with each other in three dimensions (3D) but do not require additional exogenous matrix or scaffold constructs [152]. Cell studies involving the use of cell lines and patient derived samples have proved to be very valuable tools in AML research facilitating insights into disease biology, drug efficacy and toxicity testing [153], and drug sensitivity and resistance tests (DSRT) in the case of patient derived blood and BM aspirates [154]. More than 1000 leukemia and lymphoma cell lines have been identified, with greater than 400 described in The Leukemia-Lymphoma Cell Line FactsBook [155]. Table 1 provides a summary of some scaffold-free in vitro models employed in AML research. Common in vitro AML cell lines studied are representative of different types of AML (acute promyelocytic leukemia cell lines, monocytic leukemia) and aggressive behavior including KG-1, KG1a, SKM-1, MOLM 13, MOLM 14, Kasumi-6, NB-4, HL-60, U937, THP-1 [156]. The cell lines may be cultured at established permissive conditions either in monoculture or in co-culture with other types of cells, e.g., native blasts, ECs, OBs or adipocytes to help characterize leukemogenesis and identify the role of other cells in the disease [157]. In a study conducted by Zeng et al. (2017), the AML cell lines OCI-AML-3 and U937 were co-cultured with mouse derived stromal cells M5 to test a combination of agents in order to target AML resistance. This study demonstrated that the mechanism of resistance is caused by signaling cues between the cells [21]. Suspension models are categorized as 3D models [158], however the lack of 3D architecture and microenvironmental cues hampers our understanding of AML and its susceptibility to drug treatment in the bone niche. As already discussed the 3D niche offers protection to leukemic cells and impacts their behavior, consequently this can influence the response to drug treatment. The lack of complex architecture has been shown to influence how the cells access nutrients [159], and other limitations include the necessary addition of exogenous growth factors to preserve the growth of primary leukemic cells [160].

**Table 1:** A summary of in vitro scaffold-free models employed in studies investigating acute myeloid leukemia

**Cell culture**	**Study goal**	**Key Findings**	**Ref**
AML cell lines (KG1a, OCI-AML-2, OCI-AML-3 and MV4-11) AML cells from patients	Verify the potential of DNTs to improve the anti-leukemic effect of common chemo- agents when used in combination and to target chemoresistant disease	Patient samples were found to be more susceptible to DNTs following a pre-treatment based on daunorubicin AraC or daunorubicin followed by DNT therapy decreased the engraftment of KG1a cells in the bone marrow of NOD.Cg-*Prkdc*^*scid*^ *Il2rg*^*tm1Wjl*^/SzJ (NSG) mice	[161]
AML samples from children (n = 132, median age 7.0 years)	Determine the clinical relevance of in vitro chemo resistance in childhood leukemia, making a comparison with leukemic cells of complete responders to treatment	In vitro resistance to the drugs can predict response to early courses of therapy	[162]
AML samples from 151 patients	Test in vitro efficacy of a panel of 66 kinase inhibitors. Proof of principle study to elaborate an algorithm to identify kinase targets and the most appropriate patient-specific therapeutic option	70% of patient samples showed sensitivity to one or more drugs. The algorithm identified the pathway dependence of cells in samples with known oncogenes and showed the in vitro test could potentially predict the clinical outcome	[163]
AML cell lines HL-60, KG-1, Kasumi-1 and primary human AML samples (blood/bone marrow)	Demonstrate how infiltration of AML cells in the BM microenvironment causes an inflammatory response by activation of the JAK/STAT pathway	The impact of some inflammatory cytokines (IL-6, IL-1β, and IL-8) on AML cells was quantified. Inhibition of JAK/STAT signaling using selective JAK1/2 inhibitor (ruxolitinib) produced significant anti-leukemic activity in vitro but lacked a substantial effect as monotherapy in a xenograft transplantation model	[164]
AML cell line HL-60 and other leukemia cell lines	Clarify the mechanisms underlying the effect of Trib1, a pseudo kinase involved in neoplastic differentiation of the myeloid progenitors, on HOXA9, a transcription factor, which regulates hematopoiesis and leukemogenesis	Trib1 overexpression induced enhancers at certain target loci such as *Erg,* a target involved in the effect of Trib1 on HOXA9 . Treatment with the BRD4 inhibitor, JQ1, inhibited the growth of AML cells in a Trib1/Erg-dependent manner	[165]

*DNT* allogeneic double negative T cells, *AraC* cytarabine, *Erg* ETS-related gene, *HOXA9* homeobox protein Hox-A9, *IL-8* interleukin 8, *IL-1β* interleukin 1 beta, *IL-6* interleukin 6, *JAK/STAT* Janus kinase/signal transducer and activator of transcription, JQ1 small molecule inhibitor of BRD4, *NSG* NOD/SCID gamma, *Trib1* tribbles pseudo kinase homolog 1

### In vitro scaffold/matrix-based models of acute myeloid leukemia

The complexity of cancer can arise due to a combination of mutations in the cell genome, but external factors in the 3D environment can also play a role. The 3D spatial environment affects how cells access nutrients, interact with the tissue matrix and with each other [157, 166, 167]. In response to the 3D environment [168], cells produce an ECM that differs in composition and quality of proteins [166] and this can have a significant impact on cellular processes, e.g., signaling, cell proliferation and tumor progression [169], malignant transformation of progenitor cells [170] and apoptosis. Particularly, in the field of blood cancer and AML, interactions between leukemic cells and the bone niche are at the heart of drug-resistance and treatment failure [171]. One of the main drivers for the development of models articulated in 3D is the need to recapitulate the biological, physical, chemical and mechanical cues experienced by cells in their native environment [172]. 3D models can be used to co-culture various cell types typically found in the environment of interest, thereby further enhancing their physiological relevance.

Indeed, 3D models can help to close the gap between in vitro cultures and the in vivo tissue environment [173] and provide a more reliable tool to predict the in vivo therapeutic outcomes [29]. Compared to animal models, 3D models offer the prospect to control extrinsic stimuli and environmental factors more tightly. Application of 3D models can potentially reduce the number of animals used in research enabling more ethical approaches to drug development by supporting the 3Rs principles of replacement, reduction, and refinement [174].

Many different 3D models are now in use and may be categorized as matrix or scaffold based and device-assisted models (e.g., rotary flasks, trans-well, bioreactors, microfluidic devices) [175]. 3D models have been designed using a variety of natural and synthetic polymers and different fabrication approaches, heavily influenced by developments in the field of tissue engineering and drug delivery [176, 177]. The evolution has given rise to more advanced 3D models incorporating multiple materials, signaling cues and cell types to recapitulate the sophistication inherent in native tissue and mimic the intrinsic biophysical, chemical, and mechanical properties. Table 2 gives a general overview of some 3D scaffold/matrix assisted models of AML with the relevant materials used. Gilchrist et al. [178] produced a maleimide-functionalized gelatin hydrogel as a testbed for hematopoietic and progenitor cells. Hydrogel crosslinking was tuned to the desirable modulus to mimic the in vivo BM microenvironment via a thiol-maleimide click reaction. Importantly, the hydrogel crosslinking approach can avoid the generation of reactive oxygen species, which can typically occur with standard light-based crosslinking approaches. Martin et al. engineered a 3D biomimetic BM environment by differentiating BM derived MSCs on porous hydroxyapatite scaffolds in a perfusion bioreactor. During perfusion, the cells deposited ECM on the scaffolds, which was subsequently used to maintain and expand HSPCs [179]. Further research demonstrated that progenitor cells from patients with AML and myeloproliferative neoplasms could be cultured for at least 3 weeks [180]. A stromal-vascular niche was generated by incorporating human adipose tissue-derived stromal vascular cells and this platform was shown to regulate leukemic UCSD-AML1 cell expansion, immunophenotype, and response to chemotherapy in a different manner compared to the osteoblastic BM niche [180]. Sidhu et al. developed a 3D synthetic PEG hydrogel in which induced pluripotent stem cells (iPSCs) were encapsulated to study hematopoietic differentiation and model transient myeloproliferative disorder (TMD), which is associated with down syndrome [181]. The PEG backbone was functionalized with cysteine-conjugated integrin-binding peptide (GRGDSPC) and crosslinked with enzymatically cleavable, bis-acrylamide functionalized peptide (GGPQGIWGQGKG). Hydrogels with polymer content ranging from 7 to 19 wt% were investigated to simulate the modulus of embryonic tissues. The low modulus (0.7 kPa) PEG hydrogel (7 wt%) was selected to model TMD. Despite the similarity in the average diameter of the iPSCs colonies between 2D and 3D, differences in aspect-ratio were reported. In 3D, the colonies appeared more spheroidal compared to a flattened appearance in 2D. The subsequent use of the 3D model for TMD studies showed a reduction in the erythroid population and a significant increase in myeloid populations in GATA1 mutant trisomic cells in comparison with disomic or trisomic lines with wild-type GATA1. These results were in keeping with TMD characteristics, suggesting the potential of the system as a cost-effective 3D model for the disease.

**Table 2:** A summary of 3D matrix/scaffold assisted models for acute myeloid leukemia

**Material**	**Cell culture**	**Key Findings**	**Ref**
Decellularized Wharton’s jelly	Monoculture of leukemia cell lines: HL60, Kasumi-1 and MV411	The interaction between the scaffolds and cells resulted in reduced cell proliferation. A change in the cell’s morphology was observed in two of the three cell lines selected: HL60 and Kasumi-1. The cells produced a higher amount of ALDH in 3D than in suspension. Positivity to ALDH was associated to the quiescent state, resistance to chemotherapy and suggested the cells express LSC-like phenotype	[192]
Calcium alginate + HA	Monoculture: Primary AML cells, K562 or HL60 cell lines Co-culture HS-5 (stromal cells) + K562 HS-5 + HL-60	AML cell lines and progenitors in the scaffold pores displayed an altered phenotype and increased resistance to imatinib and doxorubicin when cultured in 3D compared to 2D	[193]
HA + collagen I	Co-culture MSCs either from patients (AML-MSCs) or h-MSCs + OBs + ECs + AML blasts	The scaffold sustained the long-term (21 days) culture of the blast cells. AML-MSCs exhibited higher proliferation compared to hMSCs In vivo implantation of the scaffold in the back of NSG mice demonstrated that AML cells proliferated in the 3D model for up to 9 months. Model demonstrated that activity of AML transduced with luciferase, was significantly reduced if the cells were susceptible to intra-peritoneal drug treatments	[194]
HA and PMMA fibrous scaffold	Mono- and co-culture HS-5 (stromal cells) + K562 (CML) HS-5 + HL-60	Sensitivity of K562 cells to imatinib is reduced when co-cultured with HS-5 in 3D compared to co-culture in 2D. A similar trend was observed for HL60 and stromal cell co-cultures treated with doxorubicin, although the result was not significant	[195]
PLGA, PU, PMMA, PLA, PCL, polystyrene scaffolds PLGA and PU scaffolds coated with collagen and fibronectin	K-562, Kasumi-6 and HL-60	Cells displayed the best growth profile over 6 weeks on PU-collagen I coated matrices in complete absence of exogenous growth factors	[196]
Polystyrene scaffolds	Mono- and co- culture of MV4-11 cells and OBs induced from MSC sourced from AML patients	Cells were pre-incubated with c(RGDfV) prior to cytarabine. Leukemia cell apoptosis reduced in the presence of OBs. c(RGDfV) disrupted adhesion and migration of leukemia cells in the matrix and enhanced sensitivity to drug treatment	[197]
Biohybrid star PEG-heparin hydrogel with matrix MMP responsive peptide sequences as crosslinkers	Mono-cultures KG1a, MOLM13, MV4-11 and OCI-AML3 Tri-cultures with HUVEC and bMSC	Cells grown in 3D and tri-culture displayed increased resistance to daunorubicin and cytarabine chemotherapy compared with 2D cultures even though cell phenotype was similar	[26]
Alginate hydrogel conjugated with RGD peptide and crosslinked to provide different stiffness	AML mono-cultures MOLM-14 and U-937. CML mono-culture (K-562)	The effect of stiffness on growth of MOLM-14 and U-937 is biphasic. Hierarchical clustering used to classify drugs into class I–III for MOLM-14 cells. Class I (ligand sensitive), class II (ligand and matrix stiffness sensitive), and class III (mechanics independent).	[189]

*AML* acute myeloid leukemia, *ALDH* aldehyde dehydrogenase, *RGD* Arg-Gly-Asp peptide, *c*(*RGDfV*) cyclo(Arg-Gly-Asp-d-Phe-Val), (*bMSCs*) bone-marrow derived mesenchymal stem cells, *CML* chronic myelogenous leukemia, *ECs* endothelial cells, (*h-MSCs*) healthy donor mesenchymal stromal cells, *HA* hydroxyapatite, *HUVEC* human umbilical vein endothelial cells, *LSC* leukemia stem cells, MMP matrix metalloproteinase, *MSCs* mesenchymal stromal cells, *AML-MSCs* mesenchymal stromal cells from AML patients, *NSG* NOD/SCID gamma, *OBs* osteoblasts, *PCL* polycaprolactone, *PEG* poly(ethylene glycol), *PLA* polylactic acid, *PLGA* polylactic-co-glycolic acid, *PMMA* polymethyl methacrylate, *PU* polyurethane

It is accepted that bioinspired 3D models fabricated from materials that resemble the natural in vivo environment and that enable temporal and spatial control over regulatory signals are superior substrates for cells to grow in and provide more accurate insights into cell behavior in terms of their morphology, proliferation, cellular cross-talk and resistance to common treatments [182–185]. However, the bone marrow is a complex environment, and it is challenging to replicate this faithfully. As previously discussed, cell behavior is influenced by environmental factors. The choice of materials and architecture design can impact cellular responses and are key considerations in modelling processes that happen within the microenvironment such as invasion or dissemination [186]. Matrix stiffness is known to regulate normal hematopoiesis [187, 188] but has also been observed to influence the growth pattern and response to drugs in different leukemia types [189]. Furthermore the added complexity of these systems means they are not as easy to use as traditional cell cultures techniques and assay techniques may require more processing to reach the endpoint [190]. Consequently, it is likely that experimental results and deductions will vary depending on construct design and experimental conditions. Also, this variability and lack of standardization across different models makes comparison of results difficult. Whilst the enhanced sophistication and in vitro culturing requirements makes 3D models relatively more expensive [191]

### In vivo models of acute myeloid leukemia

Despite progress in materials science and fabrication methods to recapitulate the BM niche and tumor microenvironment in 3D, and the obvious benefits these models confer compared to 2D counterparts, the use of animal models including mammalian, e.g., mice and non-mammalian, e.g., Zebrafish and Drosophila represent powerful preclinical tools to investigate the physiological and molecular cues of leukemogenesis, and also to screen potential new drug substances, and have been reviewed elsewhere [151, 198, 199]. Rodent models have played a fundamental role in advancing our basic understanding of cancer biology including disease aetiology, the interaction between the disease and host and the role of the microenvironment [200]. They are routinely employed in drug development studies to understand the pharmacokinetic, pharmacodynamic and toxicology properties of novel therapeutic agents [201]. Additionally, they have been used to model resistance and to discover biomarkers that assist in predicting disease response and progression [200]. Crucially, rodent models are advantageous because they represent the complexity of whole organism systems. Brown Norwegian myelogenous leukemia (BNML) are transplantable leukemia rat models and can be induced with promyelocytic leukemia by exposition to chemicals such as dimethylbenzanthracene [202]. The BNML rats have been shown to share some similarities with human AML in progression and pathology [203].

#### Mouse models

Mice are popular models owing to their relative genetic similarity with humans, their small size, dependable breeding, availability and relatively low cost [204]. The availability of the human and mouse genome sequences and the capacity to modify the mouse genome have also made it an attractive model in AML research [205]. However, interspecies differences and idiosyncrasies of the murine model need to be considered. Optimal features in the case of drug discovery include low cost, good penetrance, short latency, representative of the human disease in terms of genetic and molecular heterogeneity, simplicity in colony management and technical use, while easily facilitating serial assessment of disease progression and treatment response in the same animal. Additionally, they should be well-defined and validated [200, 204, 206].

Different strategies exist for modelling AML, and these have been classified as spontaneous, xenograft, syngeneic mouse models, genetically engineered murine models (GEMMs), and humanized models. These have been reviewed in detail elsewhere [207]. Selected examples are presented in Table 3. Spontaneous models develop the disease idiopathically but can be triggered by external factors including exposure to viruses, irradiation or chemical substances [207]. In early animal models disease induction involved IV injection of tumorigenic substances (e.g., aromatic hydrocarbons or N-methyl urea) [208, 209] or by application of ionizing radiation [210].

**Table 3:** Selected examples of murine models used in acute myeloid leukemia research

**Model description**	**Study observations**	**Ref**
*PML/RARα* conventional model. Human PML/RAR*α* cDNA cloned into hMRP8 expression cassette. Transgenic animals generated from FVB/N mice	PML/RAR*α* fusion protein produces APL. Leukemic cells transplanted into non-irradiated FVB/N mice demonstrated the malignant character of the myeloid leukemia.	[245]
Retroviral model. Bone marrow LKS^+^ were transformed with a retroviral MLL-AF9 construct. Serial transplantation of AML cells from Terc^-/-^ mice into syngeneic irradiated secondary recipients to assess long-term LSC function	Deletion of the telomerase subunit Terc induced cell-cycle arrest and apoptosis of LSCs in a retroviral mouse AML model.	[246]
NRAS/BCL-2 transgenic model generated by crossing MRP8hBCL-2 with MRP8NRASD12 hemizygote mice in FVB/N strain. MDS progresses to AML	Treatment with BCL-2 homology domain 3 mimetic inhibitor, ABT-737 prolonged survival. Transplantation of cells from treated mice to lethally irradiated secondary recipients led to increased survival.	[247]
*NPM1c*^+^ + *FLT-3-*ITD compound transgenic model generated by crossing conditional Npm1^*flox − cA/*+^ with constitutive Flt3^*ITD/*+^ to generate double heterozygous mice, subsequently crossed into Mx1-Cre transgenic mice	Compound transgenic model yielded AML after a short latency. However, no case of AML was observed in Npm1c, Flt3-ITD single mutant or WT mice	[227]
Transduction of granulocyte macrophage progenitors using MSCV viral vector and transplantation	Study investigated the potential of the MLL-AF9 fusion protein to transform committed progenitors to LSC. Transplantation of transduced cells into syngeneic mice produced AML	[231]
MN1 transgene. Transduction of CMP and granulocyte-macrophage progenitors using MSCV viral vector and transplantation into irradiated mice	Transformation induced in CMP, but not granulocyte-macrophage progenitors by MN1. MN1-leukemogenicity requires MEIS1/AbdB-like HOX-protein complex	[248]
Reversible transgenic model for inducible expression of human MLL-AF9 based on rtTA	Study investigated cell of origin (LT-HSC and granulocyte-macrophage progenitors) effects on iMLL-AF9. MLL-AF9 expression in mouse LT-HSCs caused invasive, chemoresistant AML	[249]
Exploit cells conditionally blocked at the multipotent haematopoietic progenitor stage to develop a MLL-r model. Clonally derived MLL-ENL involved transduction of Hoxb8-FL cells with pMSCV-MLL-ENL-IRES-eGFP retroviral vector. Cells were cultured in MC and subsequently in IL-3. Transformed cells were injected in the tail of lethally irradiated mice	MLL-ENL driven model of AML to understand early leukemogenic disease development. Mice transplanted with MLL-ENL transduced cells developed AML within 75-days	[250]
Immunocompetent allograft model. AML cells carrying the MLL-ENL translocation transplanted by tail vein injection in C57BL/6/ BrdCrHsd-Tyrc mice	Therapeutic potential of ATR kinase inhibitors in MLL-driven AML investigated. Median lifespan of animals was increased from 23 (vehicle) to 33-days using a treatment protocol (treatment started day 13). Median survival increased to 45-days in mice on a “prevention” protocol (treatment initiated on the day AML cells were injected)	[251]

*AML *acute myeloid leukemia*,* *APL* acute promyelocytic leukemia, *CMP *common myeloid progenitors, *FLT3-ITD* fms-like tyrosine kinase 3 internal tandem duplications, *FVB/N* friend virus B strain, *iMLL-AF9* inducible expression of the human MLL-AF9, *LSCs* leukemia stem cells, *LT-HSCs* long-term hematopoietic stem cells, *MC* methylcellulose, *MLL* mixed lineage leukemia, *mRNA* messenger RNA, *MSCV* murine stem cell virus, *MDS* myelodysplastic syndrome, *NOD* non-obese diabetic, *NPM1* nucleophosmin 1, *PML* promyelocytic leukemia gene, *PML-RARa* promyelocytic leukemia/retinoic acid receptor alpha, *rtTA* reverse tetracycline controlled transactivator system, *SCID* severe combined immunodeficiency, *WT* wild-type

Xenograft studies have been employed to hasten the drug development process due to their ease of use and low cost. Xenograft models can be sub-divided into patient-derived (PDX) and cell line derived (CDX), and are typically developed by transplanting human tumor tissue or cancer-derived cell lines, respectively, subcutaneously or intravenously into immunodeficient mice including severe combined immunodeficiency (SCID), non-obese diabetic (NOD), nod-scid gamma (NSG) mice [189, 211, 212]. In the context of hematological cancers, variations on this approach have involved orthotopic injection of cells into the animal’s bone marrow [200]. These models are regarded to have several deficiencies, which has been reviewed elsewhere [200], including defects in the immune response of SCID and nude models and imperfections in DNA repair in SCID mice. Notably subcutaneous xenograft models  can oversimplify the disease process by failing to mimic the complexity, ongoing changes and physiological interactions between the host stroma and malignancy and do not completely capture the regulating effects of the microenvironment in studies evaluating drug response [213]. Orthotopic transplantation into the tissue of interest can go some way to addressing this drawback [204]. Cell line-derived xenograft models are typically generated using a few human cells lines and do not reflect the genetic diversity inherent in patient populations, which makes PDX superior in this regard. Added to this CDX models are limited by poor predictive ability in the clinic [214, 215]. While the ability to collect samples from patients at different stages of their disease and treatment underscores the greater utility and increasing popularity of the PDX compared to CDX models [216]. However, the use of immune deficient animals in CDX and PDX models can limit their use in preclinical studies designed to evaluate immune-oncology drug-therapies [217]. This is especially noteworthy given the increased focus on immune-oncology and the number of candidates in the clinical pipeline for AML, and other cancers.

Genetically engineered mouse models induce the disease by genetic manipulation of the DNA [218], and offer the prospect to imitate the genetic and biological progression of the disease in human counterparts and have been the mainstay of basic cancer biology research in the last few decades [217]. Mice can be genetically engineered to express dominant oncogenes in the mouse germline or by mutation of tumor suppressor genes. AML was amongst the earliest diseases to be modelled using transgenic animals owing to its relative genetic simplicity [219, 220]. Transgenic models can be produced by several methods and have been reviewed elsewhere [221]. Different types of transgenic models exist including knock-out mice where deletion or silencing the gene of interest can cause loss of function and knock-in models that can add an altered gene version have been used to study oncogene overexpression [222, 223]. Constitutive models have been valuable in cancer research but do not recapitulate the sporadic development of disease and constitutive random insertion models can result in undesirable expression levels, lack of tissue specificity and lethality [221]. Engineering advances led to the production of conditional models that allow the temporal and spatial expression of the gene of interest in response to a modifier and inducible models where transgene expression is under the control of specific drugs, e.g., doxycycline [222]. Conditional models include the Cre-loxP and *Flp* recombinase recognition target site (Flp-Frt) systems [206, 224–226]. Compound transgenic/knock-in mouse models have been generated to reflect disease complexity and to study the requirement for cooperating mutations in AML [207]. The combined effects of two commonly occurring somatic mutations in AML Nucleophosmin 1 (NPM1) exon 12 mutations (NPM1c) and internal tandem duplications of *FLT3* (*FLT3-ITD*) were examined by crossing conditional Npm1^*flox − cA/*+^ with constitutive Flt3^*ITD/*+^ to generate Npm1^*flox − cA/*+^; Flt3^*ITD/*+^ double heterozygous mice, which were then crossed into *Mx1-Cre* transgenic mice to induce recombination of Npm1^flox − cA^ in HSCs. The study revealed a strong molecular synergy with the development of AML after a short latency [227].

Furthermore, reversible, temporal transgene expression has been accomplished using tetracycline-inducible transgenic systems to generate induction or repression of the target gene in response to tetracycline [228]. The two basic variants of tetracycline-controlled gene expression include the Tet-off system and the Tet-on system. The systems consist of two elements, the Tet operon promoter (Tet-O) that regulates the gene of interest and a transactivator, either transactivator (tTA) or reverse transactivator (rtTA) [228]. Gene expression is repressed in the Tet-off system, whereby the presence of tetracycline prevents the tetracycline-controlled tTA from binding to Tet-O [229]. While in the Tet-on system, the rtTA requires the presence of tetracycline or its derivatives for specific Tet-O binding and gene expression [230]. These models are useful because they allow the serial induction and repression of the gene of interest by withdrawing and adding tetracycline analogues, e.g., doxycycline to the animal’s drinking water [200].

Some of the key limitations associated with transgenic mice include the time, effort and cost required to generate new transgenic mouse models [221]. Additionally, some approaches can lead to phenotypic variability necessitating increased numbers of animals to generate the target model [206]. These drawbacks have helped drive advancements in methods to generate transgenic mice including different methods to modify DNA and non-germline GEMM (nGEMM), which have genetic modifications in some of the somatic cells but not in the germline cells [206]. nGEMM have been generated using chimeric or transplantation models. Mouse in mouse transplantation models involve isolation and in vitro transduction of murine HSPC using retroviral vectors or genome editing followed by intravenous transplantation into irradiated recipients. The donor derived transformed cells can dominate the host haematopoiesis eventually leading to leukemia [207]. Committed granulocyte–macrophage progenitors were transformed by introducing MLL-AF9 fusion protein encoded by t(9;11)(p22;q23) using murine stem cell virus (MSCV). AML developed within 80 days of injection into sublethal irradiated syngeneic recipients [231]. Transplantation models have provided important insights into the in vivo transforming potential of genetic aberrations associated with AML and some research has shown that these mutations on their own do not induce AML [207].

Other DNA modification techniques have been investigated to increase reliability and speed of gene testing. Commonly used methods include transposon-based insertional mutagenesis, RNA interference and engineered nucleases. Transposon-based insertional mutagenesis involves DNA sequences called transposons that can move from one location on the genome to another. Two groups of transposons exist including retrotransposons and the more promising DNA transposons [232]. DNA transposons are used in conjunction with transposase enzymes, which identifies specific DNA sequences and “cuts” the DNA between them. The removed sequence is mobilized and re-integrated at another site in the genome. Two effective transposon systems for insertional mutagenesis exist including include *Sleeping Beauty* and *PiggyBac* and have been described elsewhere [232]. These differ in terms of the size of the cargo, transposition activity, insertion site and the presence of footprint after transposon excision [221]. Activation of a humanized Npm1c knock-in allele in mouse hemopoietic stem cells caused Hox gene overexpression, enhanced self-renewal and expanded myelopoiesis. However, only one third of mice developed AML. This, together with a long latency highlighted a potential need for cooperative mutations. Sleeping Beauty transposon technology was used to identify cooperative mutations, which caused rapid-onset AML in 80% of mice with Npm1c [233].

More recently, novel tools have been investigated to modulate DNA including the transcription activator-like effector nucleases (TALEN) and clustered regularly interspaced short palindromic repeats (CRISPR) [234]. In particular CRISPR/Cas mediated genome engineering [235] has been proposed to recapitulate the genetic complexity inherent in human malignancies and to address the challenging and time-consuming limitations associated with traditional gene targeting [236]. A detailed review of the development and applications of this technology for genome engineering are reviewed elsewhere [237]. Greater than 85% of adult AML patients have mutations in two or more driver genes underscoring the need for new representative models that reflect the combinations of mutations inherent in human disease [238]. CRISPR/Cas technology is attractive due to the potential to generate mice carrying mutations in multiple genes in a more efficient manner compared to sequential recombination in ESC and inter-crossing of mice with single mutations [239]. Seminal work in genome editing AML-associated mutations involved delivering combinations of small guide RNAs (sgRNAs) and Cas9 with a lentiviral vector [240]. Up to five genes in a single mouse HSC were altered resulting in clonal outgrowth and myeloid malignancy. AML models with cooperative mutations mimicking the combinations of mutations observed in patients comprised genes encoding epigenetic modifiers, transcription factors and mediators of cytokine signalling [240]. Another study utilized a ribonucleoprotein based CRISPR/Cas9 system to mediate multiplex gene editing of murine hematopoietic progenitor cells, followed by transplantation into irradiated recipients. Sequencing experiments showed clonal expansion that progressed to AML in some recipient mice, while some developed hematopoietic failure. Other mice died from severe anemia, although serial transplantation with whole bone marrow cells suggested that the edited cells from these animals had not fully transformed to AML [241]. CRISPR/Cas is a powerful and versatile technology that has been widely adopted in research, however it does present limitations in terms of variability in editing efficiency and specificity [242, 243]. Research is ongoing to address these drawbacks and to categorize the activity and specificity of Cas9 variants [244].

#### Zebrafish

Zebrafish is a non-mammalian model and it has been used in cancer research for many years as an alternative to common mouse models [151]. This model has been well-established and widely used for studying the hematopoietic system [252]. In the field of leukemia, it has been used as it shares multiple pathways and transcription factors of the hematopoietic process with mammals [253]. However, some differences still exist. For example in Zebrafish, HSCs reside in the kidney marrow, which shares similar functionality to the mammalian bone marrow [254]. Other differences include the lack of lymph nodes [255] and the fast development and early dependence on the innate immune system [256]. Multiple models of Zebrafish have been created. A human oncogene MYST3/NCOA2, otherwise referred as MOZ-TIF2, was generated by Zhuravleva et al. 2008 under the Zebrafish promoter sp1/pu.1 and caused AML after 14–26 months [257]. This first model of Zebrafish was based on the inv(8)(p11;q13), a genetic alteration, which has been observed in some patients diagnosed with AML [258, 259]. Other currently used models include Spi-1: MYST3/NCOA2-EGFP, the name indicates all the mutations to generate a fusion protein, which targets a myeloid promoter; or hsp70: AML1-ETO that replicates the chromosomal mutation translocation occurring between chromosomes 8 and 21 and ends with the physiological accumulation of non-circulating hematopoietic cells, neutrophils and immature hematopoietic blasts in the intermediate cell mass of the model [151]. Indeed, multiple models can be generated, as many different types of AML arise. This, together with lower costs, quick development of embryos, the possibility to undertake population studies, to conduct in vivo imaging of HSC generation and differentiation, and the reproducibility and high efficiency in transgenesis make Zebrafish a valuable alternative to other in vivo models [254, 260, 261].

### Patient-derived samples

**Fig. 4:**
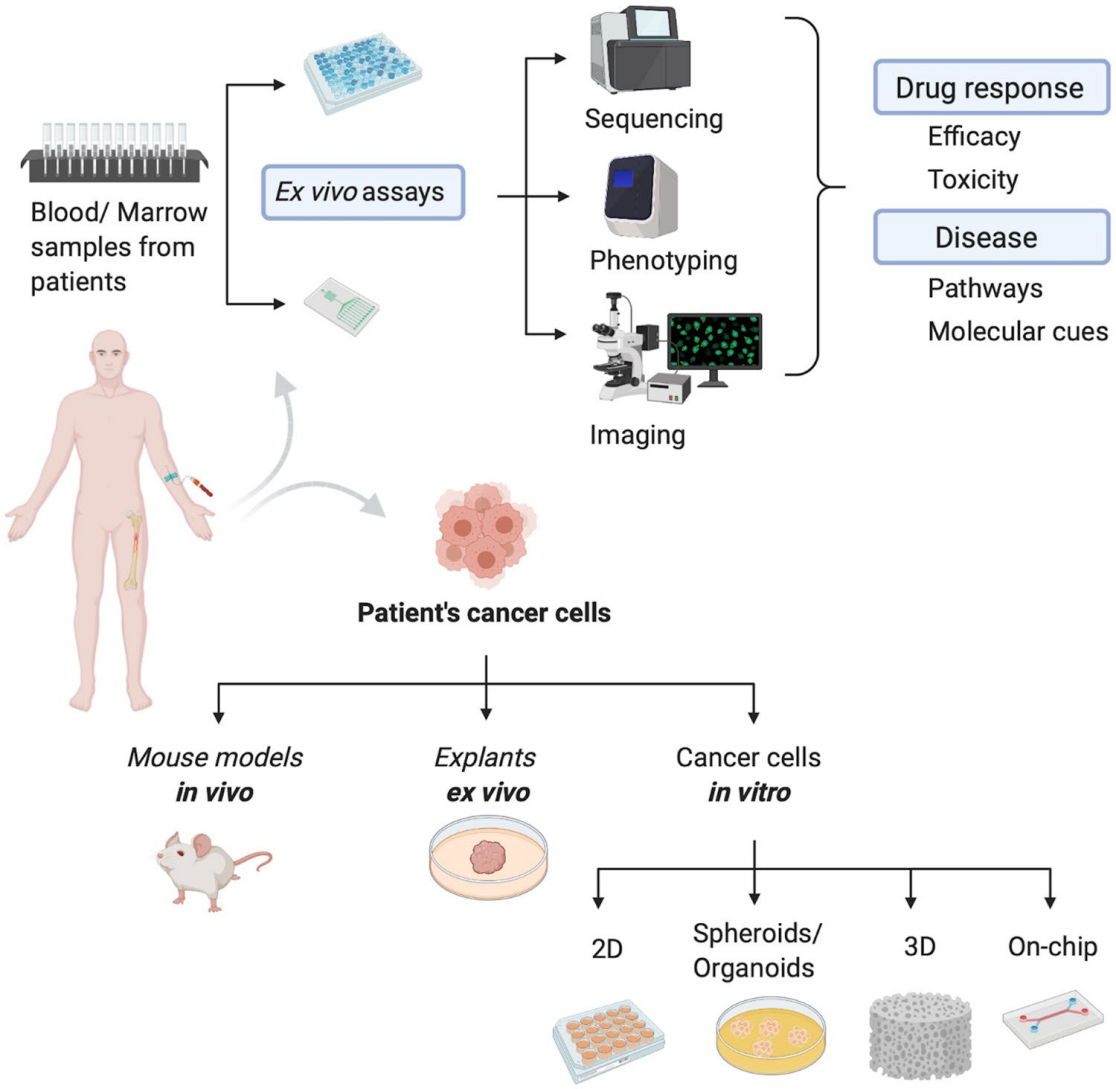
Schematic representation of the different approaches to study samples from patients. General examples of typical workflows depending on starting material including analysis of patient blood or marrow samples (**Top**). Patients’ cancer cells can be isolated from the samples and used as the starting material to undertake personalized cancer studies by establishing in vivo, ex vivo and in vitro models which include common and novel approaches like the use of organ-on-chip devices (**Bottom**). Created with BioRender.com

There is a need for models that represent the inherent variability of AML and to integrate a more patient centric approach to the development of selective and targeted drug therapies [262, 263]. Patient derived samples are used in several ways to create representative disease models and in drug screening as represented in Fig. 4. They represent a more clinically relevant model and help address many of the limitations inherent in transformed cell lines utilized in drug discovery research [264], and are important in helping to advance the field of precision oncology [265]. However, robustness, flexibility and scalability challenges associated with in vitro primary cell-based research techniques have limited their wide scale adoption in early drug development studies [266]. The use of patient samples together with advancements in next generation sequencing technology and drug sensitivity/resistance profiling are being investigated to optimize safe and personalized cancer therapies and to determine the therapeutic effectiveness of novel therapeutics [267, 268]. Pemovska et. al. developed an individualized systems medicine approach that utilized molecular profiling and ex vivo DSRT of 187 approved and investigational oncology drugs using samples from 28 AML patients [269]. This approach enabled clinically actionable drugs to be identified on a personalized basis and consecutive sampling from patients provided insights into disease evolution, drug resistance and disease management. Pharmacoscopy is a recent concept introduced by Snider et al. that allows the screening and quantification of the markers of interest expressed by individual blood cancer cells and identifies the specific response of individual cells to the panel of drugs administered. It represents a DSRT methodology capable of predicting the patients’ drug response following the analysis of data collected from automated, microscopy-based methods carried out on ex vivo samples from patients with hematological malignancies [270]. This multi-step approach is still under evaluation, but preliminary results from a retrospective study conducted on 20 patients diagnosed with AML, showed the methodology was able to predict the clinical response with an accuracy of 88.1% [270].

Primary AML cells have also been used to evaluate novel therapeutics. In one study, cells from six newly diagnosed adult AML patients were used to study antibody targeted cyclodextrin-based nanoparticles in single and combined therapy with Ara-C in vitro. The selective delivery of bromodomain-containing protein 4 (BRD4) siRNA to AML cells was achieved by targeting the surface antigen CD123 and resulted in downregulation of mRNA and increased apoptosis of leukemic cells [88]. Several studies have also been conducted to reflect the important dynamic between leukemic cells and their interaction with the BM stromal environment. Co-culture of patient derived samples with BM stromal cells has been undertaken to understand the factors that promote leukemic cell survival and chemoresistance. One study investigated inhibition of transforming growth factor beta 1 (TGF-β1), which is produced by stromal cells and implicated in cell proliferation and survival, using the antibody-based inhibitor, 1D11. Ex vivo primary AML cells co-cultured with MSCs explanted from healthy donors demonstrated that blockade of TGFb with the antibody-based inhibitor further enhanced cytarabine (Ara-C) induced apoptosis of AML cells in normoxic and hypoxic conditions [271], the latter is reputed to contribute to leukemic cell survival [272].

Patient-derived samples have also been employed to create PDX and hybrid models. Different approaches describe isolation of the cells of interest from ex vivo samples (e.g., blood and marrow) sourced from healthy, untreated and newly diagnosed AML patients and subsequent establishment of in vivo xenograft models, Fig. 4. In a bid to recapitulate the disease condition more closely, a leukemia niche xenograft model was established by first seeding a 3D ceramic scaffold with human MSCs prior to implantation into female NSG mice [273]. Eight weeks after implantation, the MSC seeded scaffolds were directly injected with blood and BM samples from patients diagnosed with AML. Histology staining with hematoxylin and eosin (H&E) on the non-injected scaffolds confirmed the presence of extramedullary bone with stromal cells and bone material within the ceramic particles. The model showed how a more ‘humanized’ environment can favor engraftment of AML samples along with a better maintenance of cell self-renewal [273]. Lee et al. (2012) established a 3D culture of human bMSCs, which were isolated from healthy human BM aspirates and expanded ex vivo prior to seeding on the surface of a collagen-coated hydrogel [274]. The scaffolds were later implanted subcutaneously in NOD-SCID IL2ry^*uill*^ mice. Four weeks after implantation, HSPC and leukemic cells were found within the scaffold structure [274].

### On-chip models of acute myeloid leukemia

The first microfluidic platform was described as a “planar” glass device with capillary channels that allowed the separation of two different dyes, fluorescein and calcein by application of electrophoresis [275]. Since their inception, significant technology advancements coupled with the ability to control fluids on the femto- to micro-liter scale have led to increased interest for a range of applications including drug screening and development [276–278]. Recent research has utilized these platforms to assess the therapeutic effects and toxicology of nanomaterials [279].Generally, on-chip devices that are used to host live cells or tissues consist of a microfluidic platform where media is perfused through the chamber containing the cell sample and waste is removed through inlet and outlet plugs. Sensors are used to control, set and sense the microenvironmental changes within the device [280]. When the technology is used to resemble either the natural cues of a particular organ or an event that happens in the organ, the on-chip technology is then called organ-on-chip technology or microphysiological systems [279, 281]. Current representations include, e.g., liver [282], lung [283], gut [284], bone marrow [285], and blood-brain barrier [286] amongst others. Indeed, an entire ‘human on-chip’ has also been reproduced [287].

These platforms enable research on a variety of physical and biochemical processes depending on the parameters studied [279]. In the field of cancer, microfluidic platforms offer the prospect to recapitulate the tumor and disease microenvironment and advance our understanding of disease pathophysiology by modeling disease processes, e.g., intravasation, extravasation, angiogenesis, invasiveness, migration, and adhesion [278, 288]. Microfluidic devices have also been used as diagnostic tools for biomarker analysis and assessment of cell sensitivity to drugs in a patient personalized manner [289], by employing devices customized with patient’s own cells [290]. This technology is advantageous compared to other in vitro models because it is possible to replicate physiological properties more accurately, e.g., hydrodynamic forces, shear forces and nutrient gradients, by setting the appropriate load volumes and flow rate. Device design is flexible, and they can be tailored to the needs of individual cell types and to create specific chemical gradients [291]. They also offer the opportunity to reduce fluid volumes in cell culture experiments [292], to create bespoke tissue environments and dynamic cell culture conditions [293], to study low numbers of cells or single cells, reduce contamination risks [291], facilitate real-time and high resolution imaging [294], conduct high-throughput experimentation [295] and significantly, can potentially reduce the use of animals in cancer research [296].

Microfluidic devices can be produced using a broad range of materials. Earlier devices were fabricated with glass and silicon, but plastics represent a more cost-effective alternative [297]. Polydimethylsiloxane (PDMS) is a first-choice material in microfluidic fabrication, due to its low autofluorescence, high permeability, biocompatibility and transparency [298]. However, drawbacks of this material include possible leaching of the plastic monomers which are a source of cell contamination, adsorption of small molecules at the surface, variability in wettability and poor compatibility with some chemicals [299]. Other technology constraints include the presence of artificial structures due to the PDMS or other plastics that reduce the overall surface area, and limit interactions between neighboring cells, limit interactions between cells and the ECM and limit exposure to soluble signals [300]. Creation of tissue specific microenvironments has involved the use of a range of materials, inspired by the tissue of interest. Selected examples are discussed later in this section.

Fabrication of the outer, PDMS microfluidic casing is undertaken using standard photolithography and soft-lithography methods [301]. Soft-lithography is common due to its availability, low cost, and high-throughput potential. Although there is increasing interest in additive manufacture (AM) techniques to produce the chip housing and biomimetic inserts to replicate the tissue microenvironment [302]. The benefits and potential of AM techniques have been reviewed elsewhere [303, 304]. In the context of microfluidic and on-chip devices, its flexibility in processing a variety of materials and the potential to create accurate and customized constructs are particularly attractive. Promising advances in AM include bioprinting of cells together with hydrogels and growth factors to enable fabrication of structures with controlled biological and mechanical properties. This offers the prospect to create 3D in vitro models that more closely replicate native tissue architecture and functionality [305]. However, 3D bioprinting is not free from challenges. Additional complexities exist owing to the need for suitable, printable, bio-inks and the technical challenges inherent with living cells [306].

Microfluidic models have been developed with a view to better understanding mechanisms underlying hematopoiesis and dysfunction and to screen new drug compounds [16]. Nelson et al. developed a multi-niche, bone marrow-on-a-chip microfluidic device by integrating a bottomless 96-well plate with a PDMS device to recapitulate the perivascular and vascularized endosteal niches of the BM [307]. MSCs were differentiated to produce a bone-like endosteal layer on the bottom surface of the device prior to seeding MSCs and HUVEC endothelial cells in a fibrin-collagen hydrogel to create a vascularized layer. This study demonstrated that the endosteal niche provided a potential protective role to HSPCs subjected to ionizing radiation, opening the possibility to explore factors influencing BM homeostasis, disease pathology and drug development [307]. Another BM-on-chip device designed to understand BM injury and recovery following exposure to stressors, such as drugs and radiation consisted of a dual-channel separated by a porous membrane [308]. One of the fluidic channels was filled with fibrin gel and was used to co-culture CD34^+^ and BM-derived stromal cells, while a parallel channel was lined with human vascular endothelium. A 2-day infusion of 5 fluorouracil through the vascular channel displayed toxicity at clinically relevant drug concentrations in line with predictions. Parallel studies utilizing suspension and static gel co-cultures did not display the expected drug toxicity at clinically relevant concentrations [308].

In the context of AML research, models that have mainly consisted of common sinusoidal microfluidic devices have been used for high throughput detection of the disease in patient samples [309]. Efforts to develop chip devices suitable for use with blood samples, which require less invasive procedures compared to BM aspirates but are limited by lower blast counts, have utilized a spiral microchip design and application of “inertial microfluidic” principles. The biochip designed to detect minimal residual disease (MRD) and enrich blast cells in blood biopsy samples of patients [310], subjects the cells in a non-linear microchannel to inertial lift and dean drag forces, allowing cells with different sizes to be isolated in specific regions in the microchannel. The device was validated by testing human blood samples from patients with different types of leukemia, including AML by optimizing the chip with the HL-60 cell line. The device was proposed to screen patients' liquid biopsy samples as an alternative to conventional flow cytometry and invasive methods owing to its enhanced sensitivity [310].

**Fig. 5:**
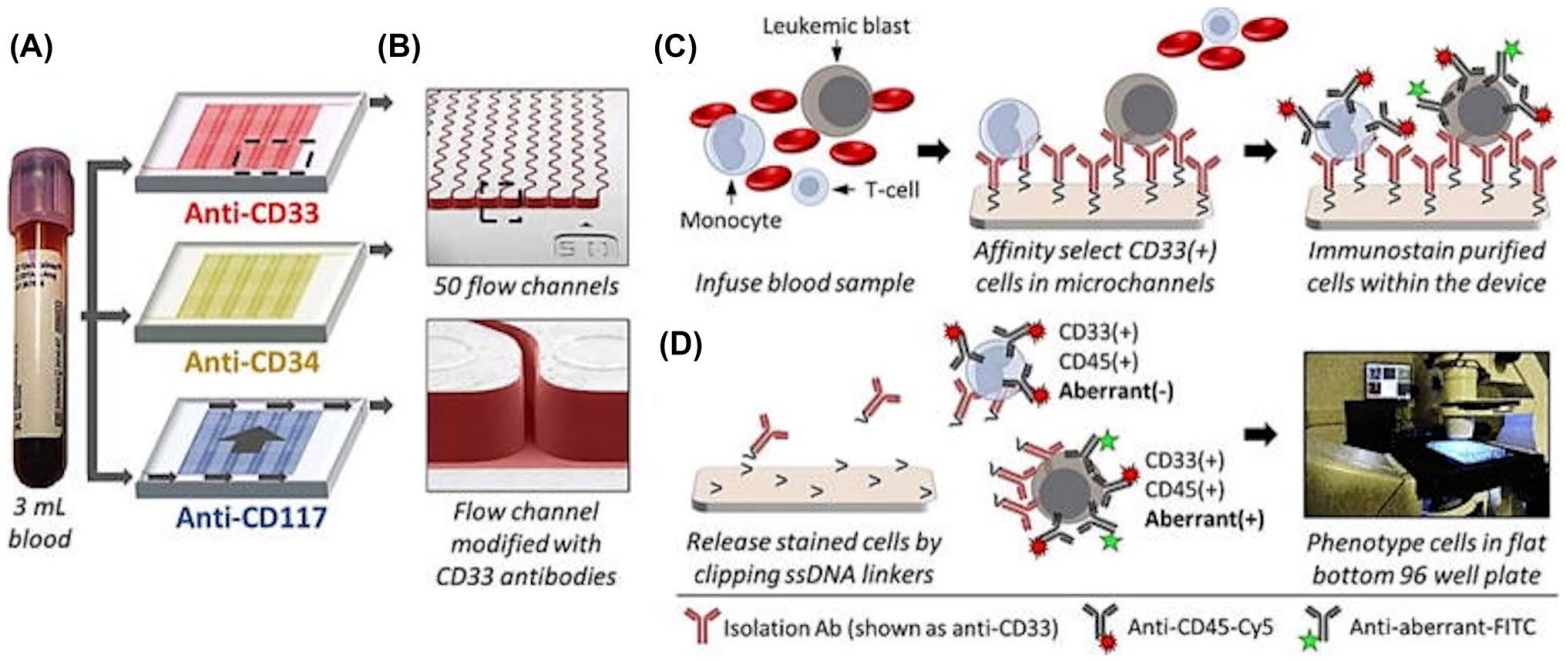
Microfluidic device used for the detection of MRD in blood samples from patients. A) The blood collected from patients is analyzed in three microfluidic devices each of those differently coated with monoclonal antibodies (mAbs) to allow the detection of leukemic cells through recognition of the surface antigens CD33, CD34 and CD117 respectively. B) Images of the 50 coated sinusoidal channels in the device, in particular the inlet of a channel. The images highlight the coating with anti-CD33 mAbs, which appear false colored in red. C) Schematic representation of the mechanisms of recognition and isolation of antigen presenting cells. CD33 + cells are selectively retained in the channels while other blood cells flow freely through the device without any retention. Selected cells are then immuno-stained followed by fixation and staining of the nuclei with DAPI. D) Details of the mechanisms of release of the retained cells from the surface of the channels. The cells are collected into flat-bottomed wells and imaged afterwards using semi-automated fluorescence microscopy. Reproduced from ref [309] with permission from the Royal Society of Chemistry

Microfluidic devices have been used to detect MRD in patient-derived blood samples by targeting surface antigens CD33, CD34, CD117 and aberrant markers CD7 and CD56 expressed on circulating leukemic cells (Fig. 5). The device was able to detect MRD at an earlier stage and was shown to be more accurate than common PCR and flow cytometry. Moreover, the approach subverted the need for patients to undergo marrow biopsy [309]. With the same aim of establishing MRD, Khamenehfar et al*.* (2016) developed another microfluidic platform. The dielectrophoretic microchip fabricated from glass using micromachining consisted of three reservoirs, a chamber to trap the cells and three electrodes. The device was used as a preclinical tool to test the cell response to daunorubicin and to detect single cell involvement in MRD [311].

Advances in tissue engineering, microfluidics and microfabrication techniques have inspired the development of sophisticated, integrated systems that move a step closer to recapitulating the native tissue environment [312, 313]. Models have been designed which consisted of 3D polymeric inserts encased in a PDMS envelope. Houshmand et al*. *(2017) developed a microfluidic chip device to test the efficacy of azacitidine and cytarabine using demineralized bone matrix as the 3D matrix in the microfluidic chamber. Co-culture of TF-1 cells with bMSCs revealed increased cell proliferation rate and drug resistance in the 3D chip model in comparison with common 2D cultures. The authors suggested it could be further applied in preclinical studies to predict in vivo therapeutic outcomes and specify the role of the AML niche in the pathology of the disease in patients [314]. Another notable example that sustained co-culture of HSPC and MSCs for 28 days in a microfluidic environment includes a 3D BM on-chip model consisting of a hydroxyapatite coated zirconium oxide ceramic (Sponceram) scaffold [19]. The scaffold designed to produce a more bio-relevant model resembling the natural architecture and molecular signaling cues inherent in the BM niche was pre-cultured for 7 days with MSCs to create a suitable environment for HSPC. Indeed, HSPC isolated from the scaffold were shown to retain their multilineage differentiation potential.

**Fig. 6:**
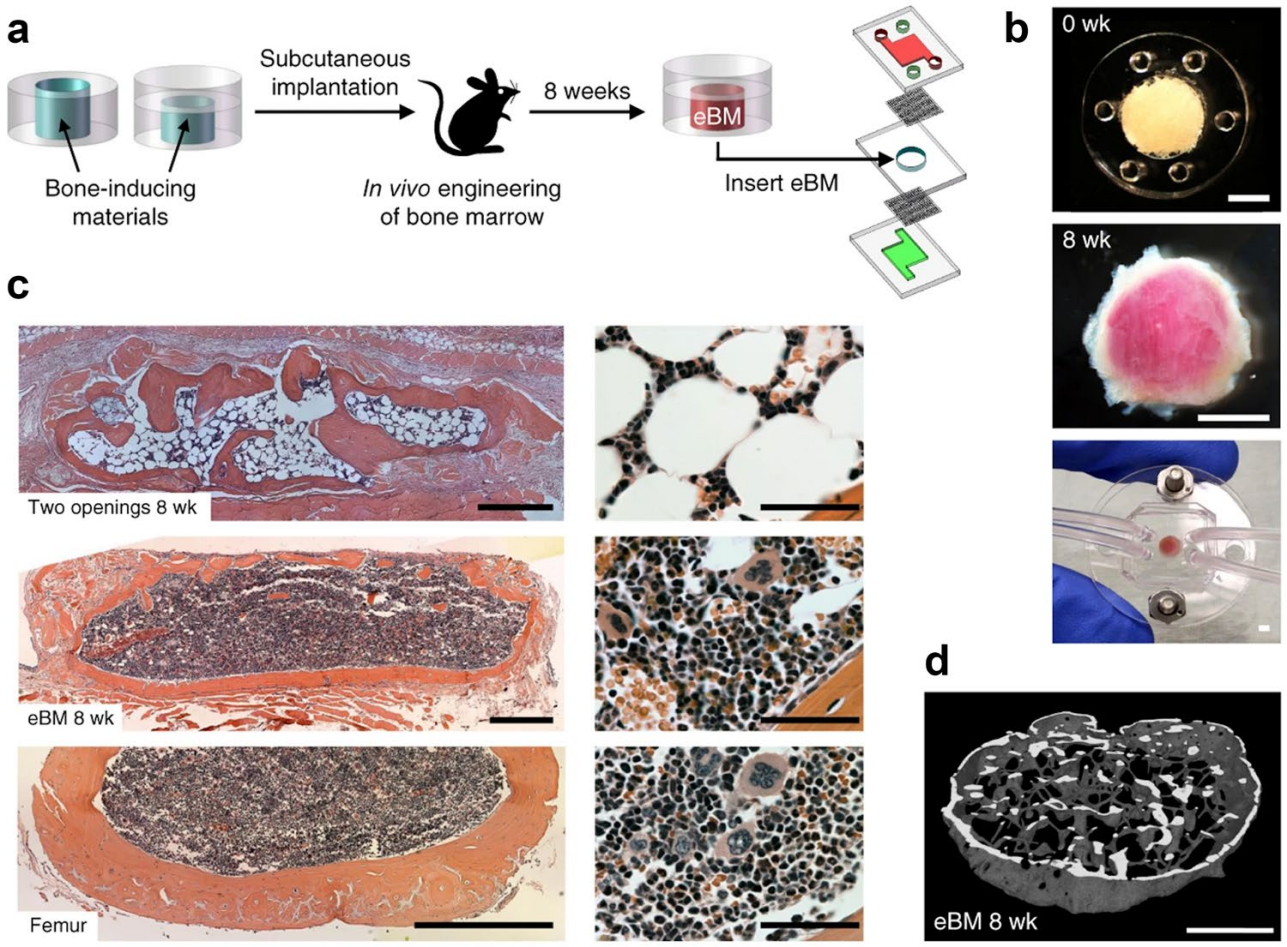
Engineered BM-on-a-chip used to grow HSPC from host. The BM on-chip consists firstly of a PDMS device (1 mm high × 8 mm in diameter), either with one or two openings, and contains a cylindrical cavity filled with bone inducing materials DBP, BMP-2 and collagen type I gel. The eBM was implanted subcutaneously in the back of a mouse for 8 to 12 weeks. After removal, the eBM was cultured in a microfluidic chip device. **A)** Schematic representation of the different steps involved in the development of the BMa-on-chip from the development and manufacturing of the eBM to the cultivation of the eBM in a separate microfluidic device. **B)** Images showing the eBM and microfluidic device. Top (prior to implantation), PDMS device with bone-inducing materials contained in its central cavity. Center (8 weeks after implantation), newly formed white bone surrounds pink marrow. Bottom, BM-on-a-chip microfluidic device used to culture the eBM in vitro. **C)** Low-left, histological images, and relative high magnification views of sections of the eBM stained with H&E in the PDMS device with two openings (top) or lower opening (center). The images are taken 8 weeks after implantation in the host. Control: cross-section of BM in a normal adult mouse femur (bottom). Scale bars, 500 and 50 μm for low and high magnification views, respectively. **D)** 3D reconstruction of micro-CT data from eBM 8 weeks after in vivo implantation (average bone volume was 2.95 ± 0.25 mm.^3^; *n* = 3). Scale bar, 1 mm.). Reproduced with permission [312] Copyright 2014, Springer Nature

Torisawa et al. 2014 developed an engineered bone marrow (eBM) model composed of a PDMS device with a cylindrical cavity filled with type I collagen gel and bone inducing materials such as demineralized bone powder (DBP) and bone morphogenetic proteins (BMP2 and BMP4) [312]. The device was implanted subcutaneously in the back of CD-1 or C57BL/6-Tg(UBC-GFP)30Scha/J mice. After colonization by the host HSC was established, the device was later explanted, inserted in a chip device and perfused with growing medium for 4 or 7 days. Histological analysis performed by staining sections of the eBM and intact femur with H&E revealed the morphological correspondence between the eBM and natural bone marrow. Micro-computed tomography (micro-CT) and energy-dispersive X-ray spectroscopy (EDS) enabled investigation of the structure and composition of the eBM, which were found to resemble the structure of a mouse vertebrae and the composition of the natural trabecular bone [312], (Fig. 6).

## Conclusions

The underlying genomic and molecular complexity which characterizes AML represents a substantial obstacle to the development of effective medicines and successful treatment of the disease. These challenges are compounded by the prevalence of the disease typically in older patients with comorbid risk factors. A better understanding of the multitude of factors impacting AML initiation and progression including cytogenetic and molecular variables have enhanced prognostic and treatment capabilities [23, 315]. However, the development of new medicines is resource intensive, and the high costs of development and attrition rates represent significant challenges for the pharmaceutical industry, healthcare providers and patients [316]. The development of a medicinal product involves several discrete stages designed to demonstrate the product’s quality, safety, and efficacy. Safety and efficacy are demonstrated in non-clinical studies and progressive clinical trials [317]. Deficits in preclinical, models mean they are failing to accurately estimate the effectiveness of novel drug compounds which is negatively impacting the number of compounds which can reach the approval stage for treatment in patients [318]. This is especially problematical when many of these failures are occurring in the more costly, later-stages of development. Enhanced screening tools could help to address the problem of late-stage failures and reduce the attrition rate of drugs in the clinical development pipeline by providing more informative, critical information at an earlier stage [316].

Standard cell suspension models play an important role in drug discovery and development in cancer research. They are easy to use, cost-effective and experiments can be conducted in controlled environments and are amenable to high-throughput however, they fail to recapitulate the complex, and dynamic environment where AML occurs [319]. This has driven increasing interest in scaffold/matrix-based 3D models which can more accurately recapitulate the pathophysiological mechanisms and the role of the BM microenvironment that are crucial to understanding the disease and developing effective drug treatments. Although the application of 3D cell culture models in the field of drug development is still at an early stage, they offer the potential to become important in vitro tools to facilitate a more efficient drug product development process whilst also addressing the animal usage burden [29]. However, before this is a viable reality in the context of AML research several hurdles remain. The exact composition and role of niche and environmental cues and the interplay between disease and environment is still unclear, which makes design of appropriate models more challenging. Research investigating the ex vivo expansion of HSC using biomaterials has shown that proliferation and expansion depends on the properties of the biomaterials and scaffolds (including physical and biochemical properties), co-culture of HSC with other cells present in the BM and environmental cues including hypoxic conditions [13]. This underscores the need to develop standardized, reproducible, and cost-effective models that are predictive and repeatable under general conditions, if this approach is to viably overcome the limitations of more primitive cell culture models [320, 321].

The use of ex vivo samples (e.g., blood or BM aspirates) addresses limitations of overly passaged and less representative cell lines. They also accommodate the compelling need for personalization in diagnosis and treatment and partially address the lack of clinical bio-relevance which is frequently cited as a limitation of in vitro representations of cancer. For example microfluidic devices combined with 3D cell cultures of patient derived samples [322] open up the possibility of personalized medicine, which is especially beneficial in the field of cancer given the fundamental heterogeneity of the disease, the propensity of the disease to continuously evolve and the variability in disease subtype and patient circumstances [19].

Given the substantial difference between humans and animals, a more cautious approach has been suggested because the use of animal models often correlates with an overestimation of the clinical potential of drug compounds [318]. The utility of animal models in particular murine models as a predictor of the human condition has long been questioned due to interspecies differences [320, 321]. Patient-derived xenograft models represent a very valuable tool in preclinical drug testing because they increase the clinical relance by reflecting the genetic diversity and phenotypic heterogeneity of the disease. However, AML can be challenging to engraft in immunodeficient mice [323]. It is important to note xenotransplantation studies have demonstrated that a subclone’s engraftment potential does not reflect its propensity to cause AML and these models may preferentially select for the growth of specific clones and indeed these models may not accurately predict clinical outcomes in drug sensitivity tests [324, 325]. Further limitations of in vivo and most preclinical studies relates to the relatively short timeframes over which experiments are run and the limited dosing schedules employed, which do not reflect the clinical situation, and make realistic estimation of clinical efficacy more difficult [324]. Despite these limitations, animal models will continue to play a fundamental role in drug discovery and understanding disease. Currently efforts are being directed to develop models that address these drawbacks and more closely resemble the human condition. Given the increased focus on immune-oncology and the number of candidates in the clinical pipeline for AML, further development of mouse models with competent immune systems and preclinical models that can predict the efficacy of novel therapeutic approaches that combine chemotherapy, targeted therapies and immune-oncology agents is warranted [217].

Novel technology approaches including Pharmacoscopy outlined in this review involve direct profiling of patients’ biopsies by immunofluorescence, automated microscopy and image analysis, and have been able to identify clinically effective therapies in a patient-specific manner with a clinical success of around 88% compared to therapeutic success of approximately 24% in the case of clinician directed regimens [326]. Advances in technology in the realm of microfluidics and fabrication approaches have also allowed the development of more sophisticated compact, 3D, high-throughput microfluidic devices, which offer the prospect to advance the current state-of-art in detecting MRD [309], screening novel drugs or defining their profile in a more precise, personalized manner [289, 327].

In summary many challenges remain. However, the burden of the disease and the costs to patients, the pharmaceutical industry and healthcare systems globally call for innovative models and approaches to provide greater insights into AML and enable the development of safe and effective therapeutic treatments for patients.

## Data Availability

Not applicable.
